# Genome-wide identification and characterization of the *bHLH* gene family and analysis of their potential relevance to chlorophyll metabolism in *Raphanus sativus* L

**DOI:** 10.1186/s12864-022-08782-4

**Published:** 2022-08-01

**Authors:** Ruihua Wang, Yuanyuan Li, Minggang Gao, Min Han, Huilian Liu

**Affiliations:** Key Laboratory of Biochemistry and Molecular Biology, Biological and Agricultural College, Weifang University, Weifang, Shandong China

**Keywords:** Basic helix-loop-helix transcription factor, Chlorophyll content, Photosynthesis, *Raphanus sativus* L

## Abstract

**Background:**

Green-fleshed radish (*Raphanus sativus* L.) is an economically important root vegetable of the Brassicaceae family, and chlorophyll accumulates in its root tissues. It was reported that the basic helix-loop-helix (*bHLH*) transcription factors play vital roles in the process of chlorophyll metabolism. Nevertheless, a comprehensive study on the *bHLH* gene family has not been performed in *Raphanus sativus* L.

**Results:**

In this study, a total of 213 *Raphanus sativus* L. *bHLH* (*RsbHLH*) genes were screened in the radish genome, which were grouped into 22 subfamilies. 204 *RsbHLH* genes were unevenly distributed on nine chromosomes, and nine *RsbHLH* genes were located on the scaffolds. Gene structure analysis showed that 25 *RsbHLH* genes were intron-less. Collineation analysis revealed the syntenic orthologous *bHLH* gene pairs between radish and *Arabidopsis thaliana*/*Brassica rapa*/*Brassica oleracea*. 162 *RsbHLH* genes were duplicated and retained from the whole genome duplication event, indicating that the whole genome duplication contributed to the expansion of the *RsbHLH* gene family. RNA-seq results revealed that *RsbHLH* genes had a variety of expression patterns at five development stages of green-fleshed radish and white-fleshed radish. In addition, the weighted gene co-expression network analysis confirmed four *RsbHLH* genes closely related to chlorophyll content.

**Conclusions:**

A total of 213 *RsbHLH* genes were identified, and we systematically analyzed their gene structure, evolutionary and collineation relationships, conserved motifs, gene duplication, cis-regulatory elements and expression patterns. Finally, four *bHLH* genes closely involved in chlorophyll content were identified, which may be associated with the photosynthesis of the green-fleshed radish. The current study would provide valuable information for further functional exploration of *RsbHLH* genes, and facilitate clarifying the molecular mechanism underlying photosynthesis process in green-fleshed radish.

**Supplementary Information:**

The online version contains supplementary material available at 10.1186/s12864-022-08782-4.

## Background

Radish (*Raphanus sativus* L.), belonging to the Brassicaceae family, is an important root vegetable crop with multiple varieties, such as green-fleshed radish. Its flesh is green due to the existence of chlorophyll, which is necessary for photosynthesis. The expression of chlorophyll biosynthesis-related genes contributes to the chlorophyll accumulation. Due to the presence of chlorophyll, chlorophyll fluorescence technology has detected the occurrence of photosynthesis in the flesh of green-fleshed radish [1].

Basic Helix-Loop-Helix (*bHLH*) transcription factor family is the second largest gene families in plant kingdom, usually classified into 15–26 subfamilies. Members of the *bHLH* gene family have been identified in many species, for example, 188 in apple (divided into 18 subfamilies, [2]), 159 in tomato (divided into 25 subfamilies, [3]) and 115 in grape (classified into 25 subfamilies, [4]). *BHLH* transcription factors have a highly conserved bHLH domain with ∼60 amino acids, which comprises a basic region followed by two amphipathic α-helices separated by a variable loop region (HLH) [5]. The basic region is relevant to the DNA binding that allows the bHLH proteins binding to the cis-acting elements in the promoter regions of the target genes, and the HLH region functions as a dimerization domain that allows the formation of homo- and/or heterodimers.

The researches show that the bHLH proteins can positively or negatively regulate the process of chlorophyll biosynthesis. Phytochrome interacting factor1(PIF1), as a bHLH protein, negatively controls the chlorophyll biosynthesis in the dark by regulating the expression of genes coding heme oxygenase (HO3), protochlorophyllide oxidoreductase (POR), and ferrochelatase (FeChII), which are involved in the chlorophyll biosynthetic pathway [6]. Ectopic overexpression of a *bHLH* gene from *Populus euphratica* enhances tolerance to water-deficit stress and results in a higher chlorophyll content and photosynthetic rate in *Arabidopsis* [7]. In *Pyrus bretschneideri*, *PbrbHLH195*-silenced seedlings have significant reduced cold tolerance and reduced chlorophyll content [8]. Phytochrome interacting factor 4 (PIF4), as a bHLH protein, activates the expression of *Nonyellowing 1 (NYE1*) gene involved in the chlorophyll degradation and restrains the expression of *Golden 2-like Transcription factor 2* (*GLK2*) gene associated with the chloroplast activity maintaining [9].

The availability of genome sequencing data has allowed the application of bioinformatics approach to analyze various gene families. However, the available information about the *bHLH* gene family of *Raphanus sativus* L. is limited. In this study, we identified the *bHLH* genes from *Raphanus sativus* L. genome and carried out phylogenetic analysis to determine evolutionary history between RsbHLH proteins and AtbHLH proteins. The syntenic analysis provided a foundation for function explore of *bHLH* genes. RsbHLH protein motifs and *RsbHLH* gene structures were also investigated. Meanwhile, the different duplication types of *RsbHLH* genes were identified. In addition, RNA-Seq results showed that the expression levels of *RsbHLHs* differed at the five development stages of green-fleshed radish and white-fleshed radish. Finally, the weighted gene co-expression network analysis (WGCNA) confirmed a few *RsbHLH*s closely related to chlorophyll content. The information from this study will facilitate gaining insight into functions of *RsbHLH* genes associated with growth and development in *Raphanus sativus* L.

## Results

### Identification and gene structure of *bHLH* genes in *Raphanus sativus* L

**Fig. 1:**
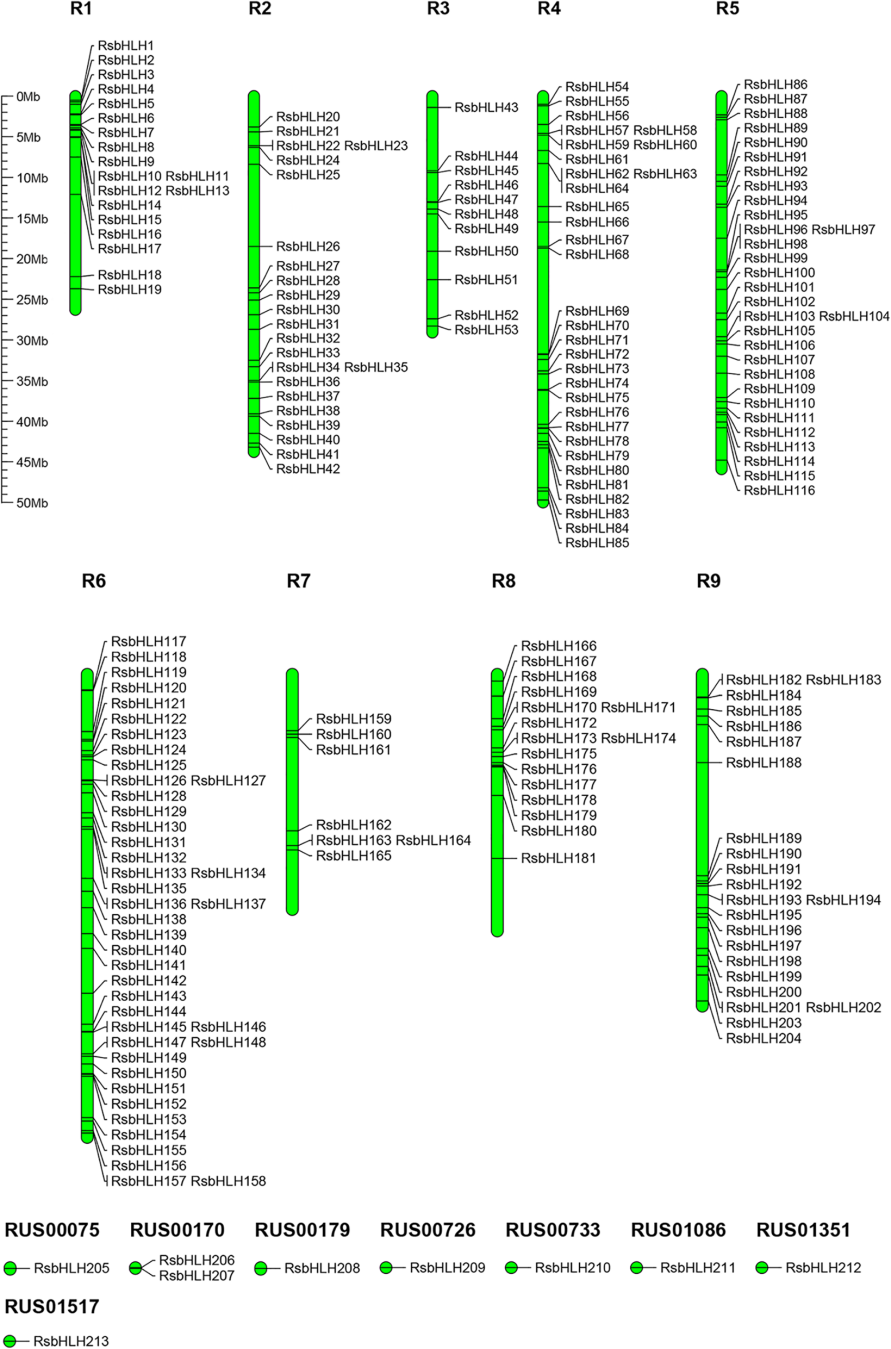
Chromosomal locations of *RsbHLH* genes

A total of 401 candidates were obtained through two searches. Subsequently, the domains of all candidates were checked to determine the existence of complete bHLH domains. Some candidates without the bHLH domain or with the incomplete bHLH domain were removed. Finally, we screened 213 *RsbHLH* genes for the further analysis (Additional file 1). A total of 204 *RsbHLH* genes were mapped on 9 chromosomes (R01-R09) according to their location information from the Radish Genome Database, and remaining 9 *RsbHLH* genes were mapped on scaffords (RUS00075, RUS00170, RUS00179, RUS00726, RUS00733, RUS01086, RUS01351 and RUS01517). Chromosome R6 had the most *RsbHLH* genes, Chromosomes R4 was next, while Chromosome R7 had the lowest number of *RsbHLH* genes (Fig. 1). All *RsbHLH* genes were renamed from *RsbHLH01* to *RsbHLH 213* based on their location on the chromosomes and scaffords.

**Fig. 2:**
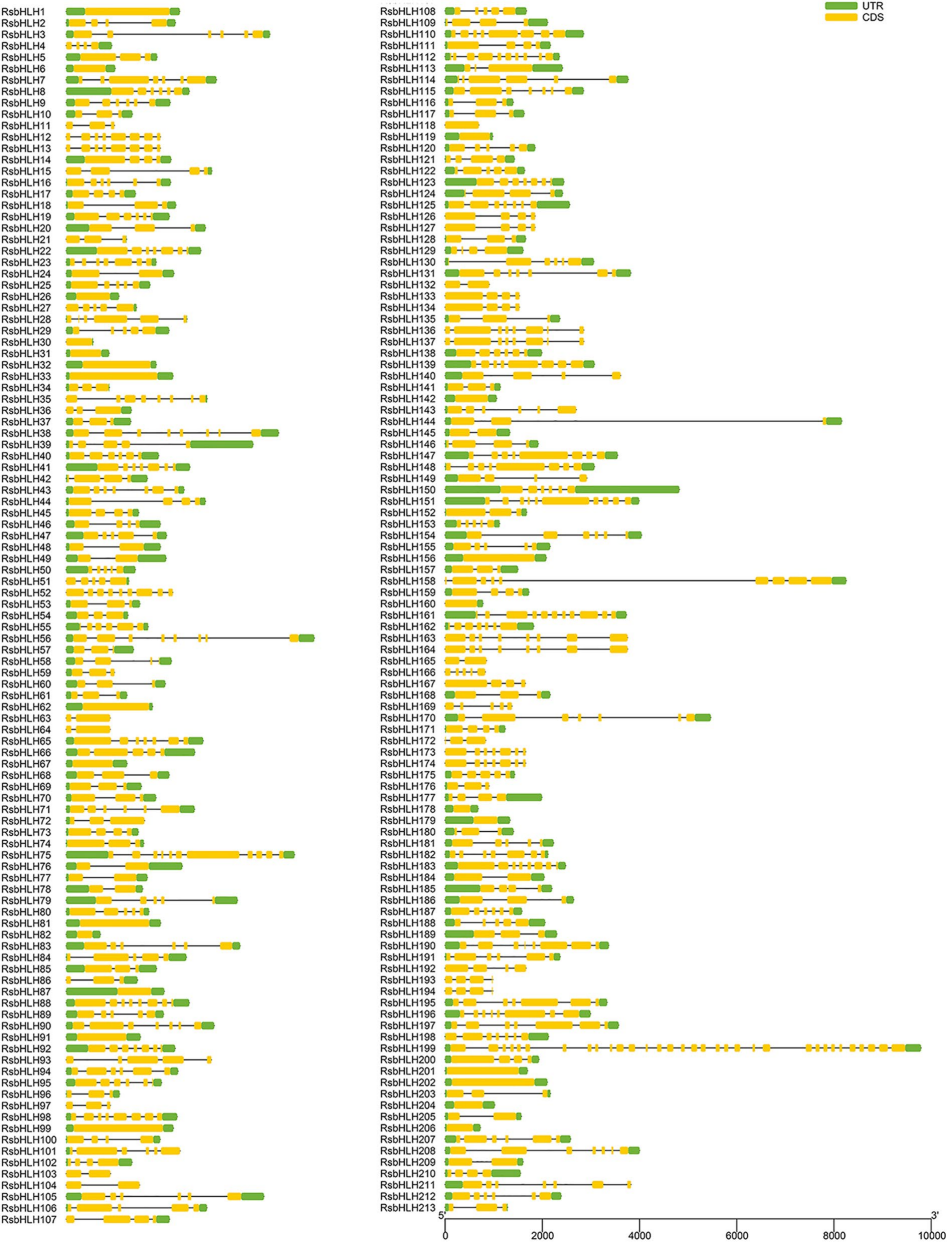
The exon–intron organization of *RsbHLH* genes. The yellow box represents the exon, the green box represents the untranslated regions (UTR), and the black line represents the intron. The sizes of exons and introns are estimated by the scale at the bottom

The exon–intron distribution of 213 *RsbHLH* members was analyzed and showed by Gene Structure Display Server (GSDS) tool (Fig. 2). The *RsbHLH* members had a varying number of exons from 1 to 33. The number of *RsbHLH*s with three exons was the largest, followed by those with four exons. There were a few *RsbHLH*s with more than eight exons. Two members (*RsbHLH52* and *RsbHLH158*) exhibited nine exons, and two members (*RsbHLH75* and *RsbHLH161*) presented eleven exons. *RsbHLH151* and *RsbHLH199* had ten exons and thirty-three exons, respectively. Additionally, 25 members were intron-less and distributed across 8 chromosomes and scafford RUS00170. The intron number of *RsbHLH199* was as high as 32, indicating that the alternative splicing form may be the most complex.

### Evolutionary tree and conserved motif analysis

**Fig. 3:**
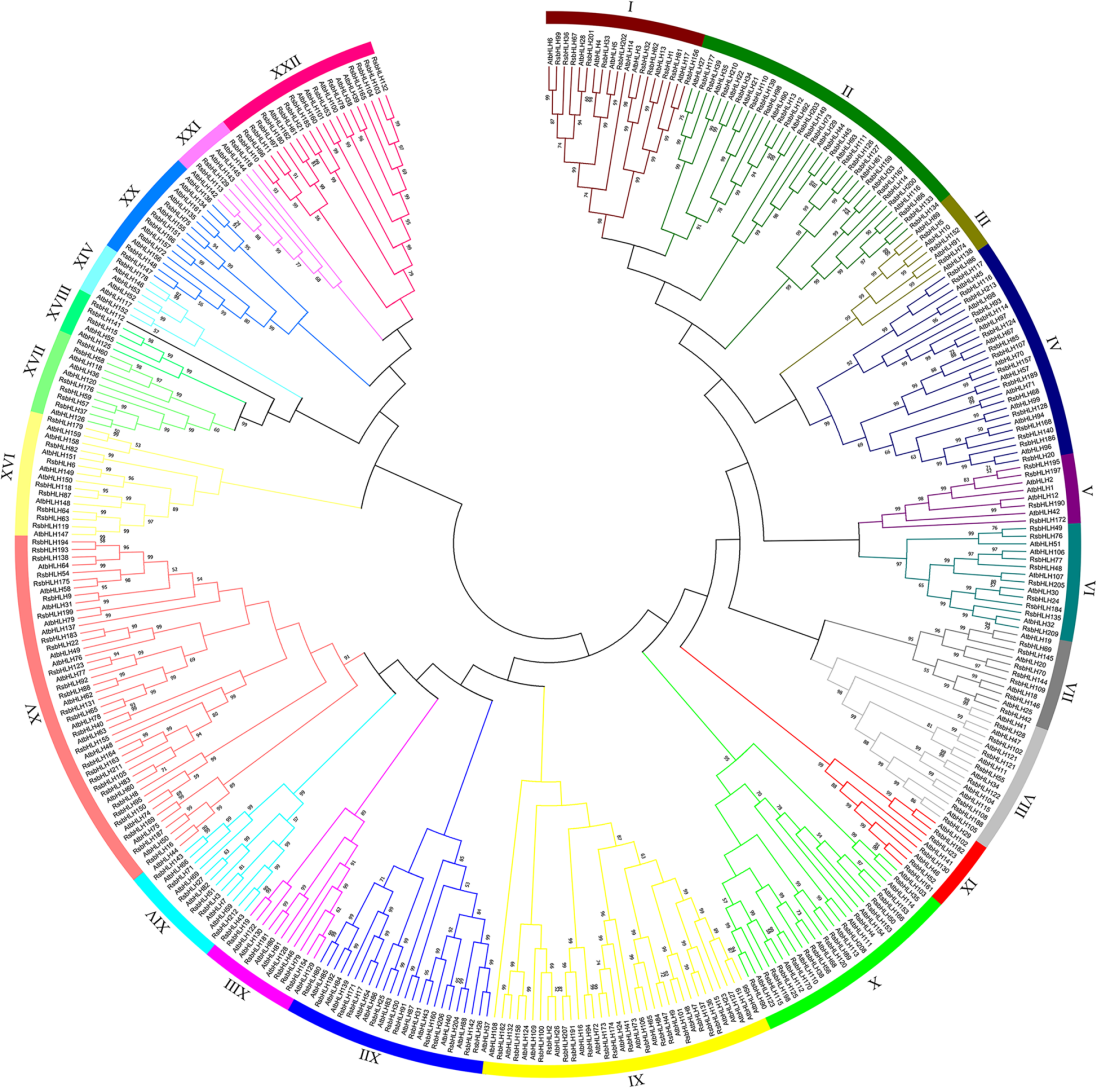
An un-rooted phylogenetic tree of the RsbHLH proteins and AtbHLH proteins. 22 subfamilies are marked with different colors

The evolutionary relationships between RsbHLH proteins and AtbHLH proteins was investigated, and a phylogenetic tree was generated using the sequences of 213 RsbHLH proteins and 158 AtbHLH protein. As shown in Fig. 3, the RsbHLH members were clustered into 22 subfamilies. The number of members in different subfamilies varied greatly, and the largest subfamily XV contained 44 members, while the smallest subfamily XVIII contained only 4 members. For the remaining twenty subfamilies, the number of RsbHLH members within each subfamily varied from 6 to 35. In the phylogenetic tree, a sister pair showed the closest genetic relationship, and the overwhelming majority of the sister pairs were orthologous pairs between RsbHLHs and AtbHLHs, such as RsbHLH65 and AtbHLH78. The studies of AtbHLH proteins were relatively clear, while the research on RsbHLH proteins was lacking. Orthologous genes usually had similar functions, so the functions of RsbHLH protein could be predicted based on the functions of AtbHLH proteins.

**Fig. 4:**
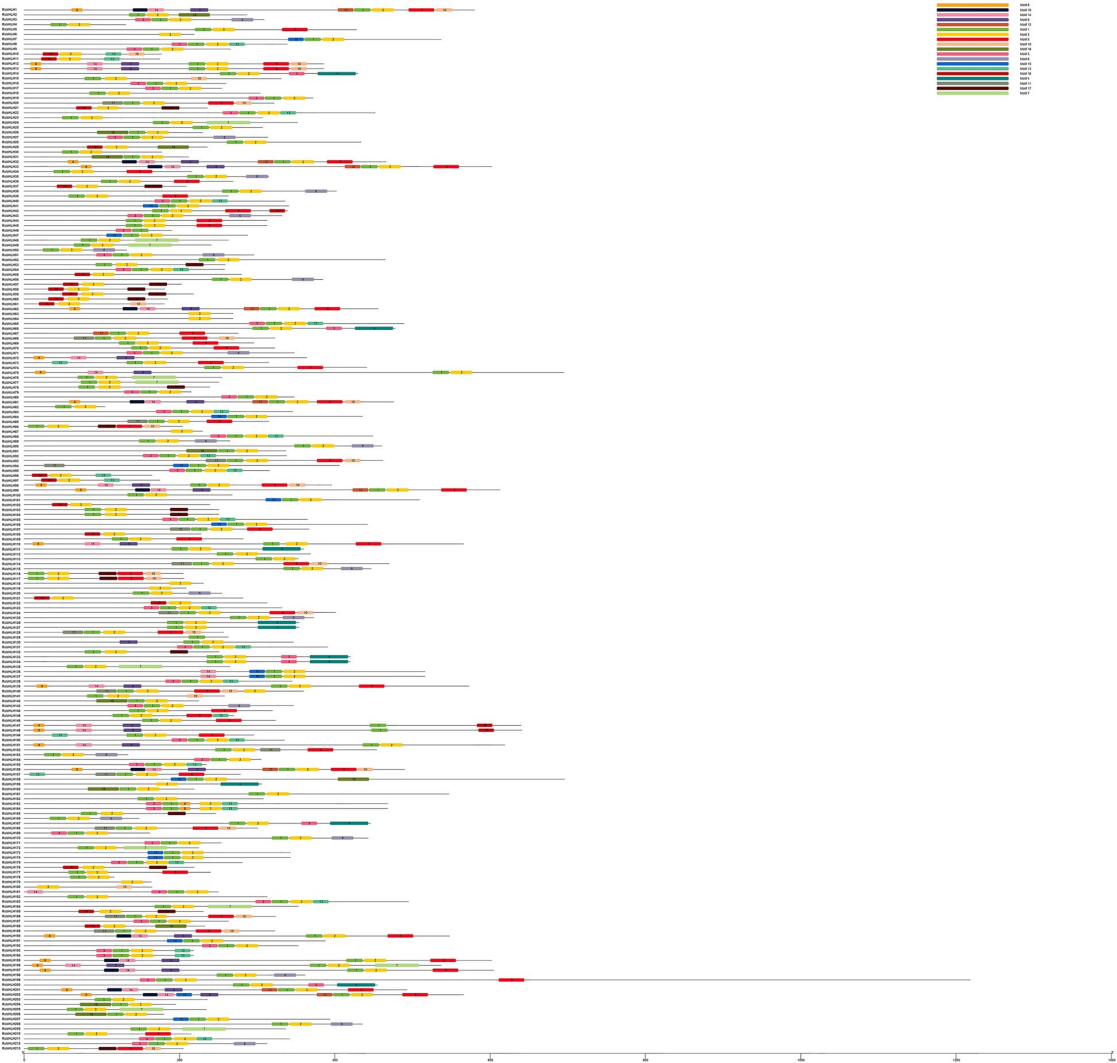
The conserved motif distribution of RsbHLH proteins. Different motifs are displayed by different colored boxes. The motif size is estimated by the scale at the bottom

The Multiple Em for Motif Elicitation (MEME) tool was employed to find out the conserved motifs in RsbHLH proteins. A total of 19 conserved motifs were identified among RsbHLHs, varying from 15 to 57 residues in length, and their logos were presented in Additional file 2. RsbHLH proteins possessed different number of conserved motifs, which ranged from one to nine (Fig. 4). Eight RsbHLHs, including RsbHLH6, RsbHLH63, RsbHLH64, RsbHLH87, RsbHLH118, RsbHLH119, RsbHLH129, and RsbHLH179, had only one motif. RsbHLH1, RsbHLH81, RsbHLH156, and RsbHLH202 had the most motifs, namely nine, while most RsbHLH proteins had three to four motifs. The Fig. 4 displayed that Motif 1 and Motif 2 were highly conserved in most RsbHLH proteins, and they were composed of 22 amino acids and 29 amino acids, respectively. In total, 16 RsbHLH proteins had Motif 1 and Motif 2 only, in addition, seven RsbHLH proteins (RsbHLH6, RsbHLH63, RsbHLH64, RsbHLH87, RsbHLH118, RsbHLH119, RsbHLH179) had Motif 2 only, and one protein (RsbHLH129) had Motif 1 only.

**Fig. 5:**
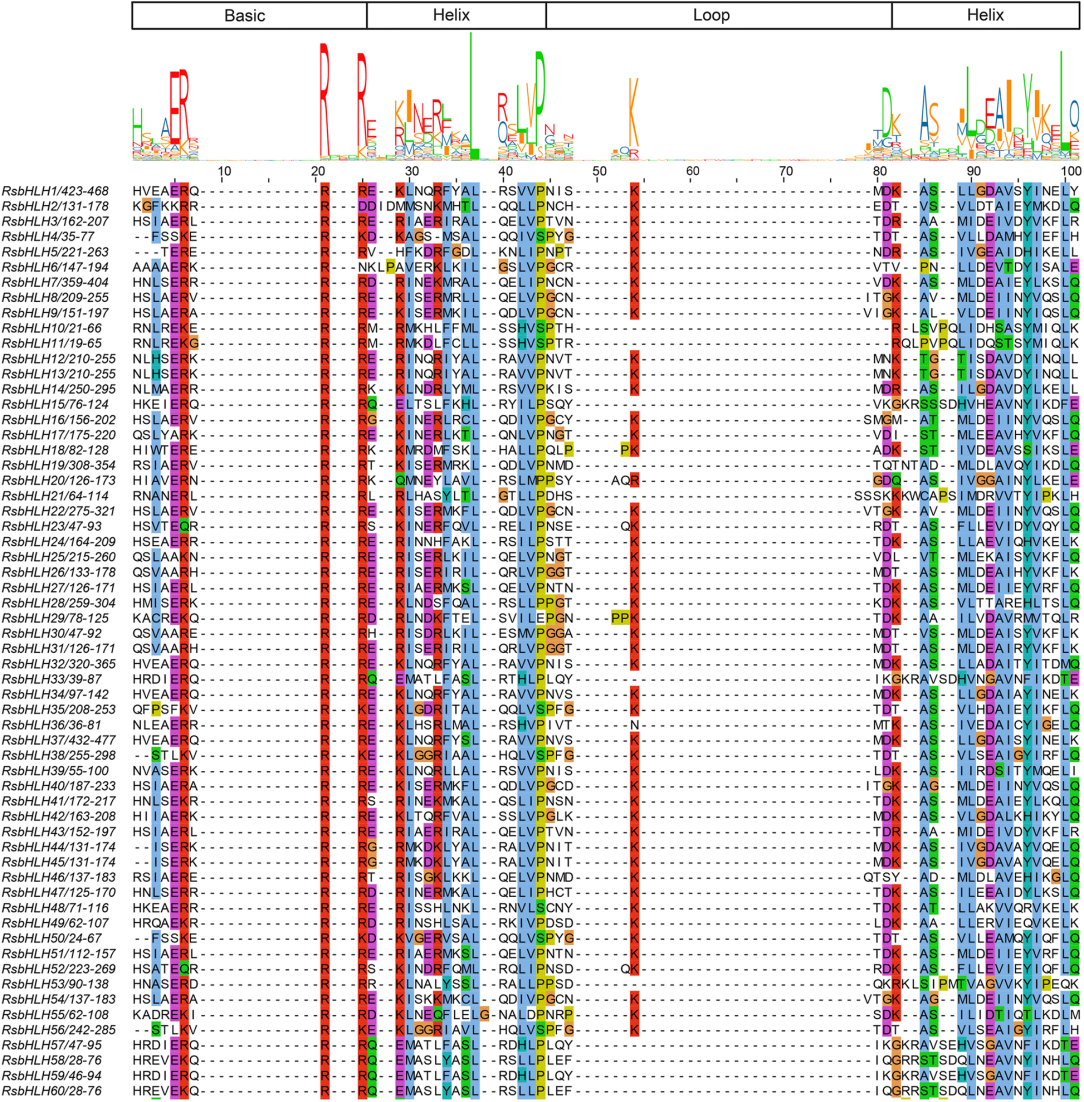
Partial display of multiple sequence alignment for RsbHLH proteins. The basic helix-loop-helix domain was displayed. The height of a letter indicates its relative frequency at the given position. This image shows only a small part of the alignment

The multiple sequence alignment of 213 RsbHLH proteins revealed that the basic region and two helix regions were highly conserved in RsbHLH proteins (Fig. 5). Among amino acids of the conserved bHLH domain, the consensus ratios of the twenty-one amino acid residues were > 50%, and seven of those were conserved with a > 75% consensus ratio. Moreover, the consensus ratios of five amino acid residues (R-21 and R-25 in basic region, L-37 and P-44 in helix 1 region, and L-100 in helix 2 region) were higher than 85%.

### Collineation and duplication analysis

**Fig. 6:**
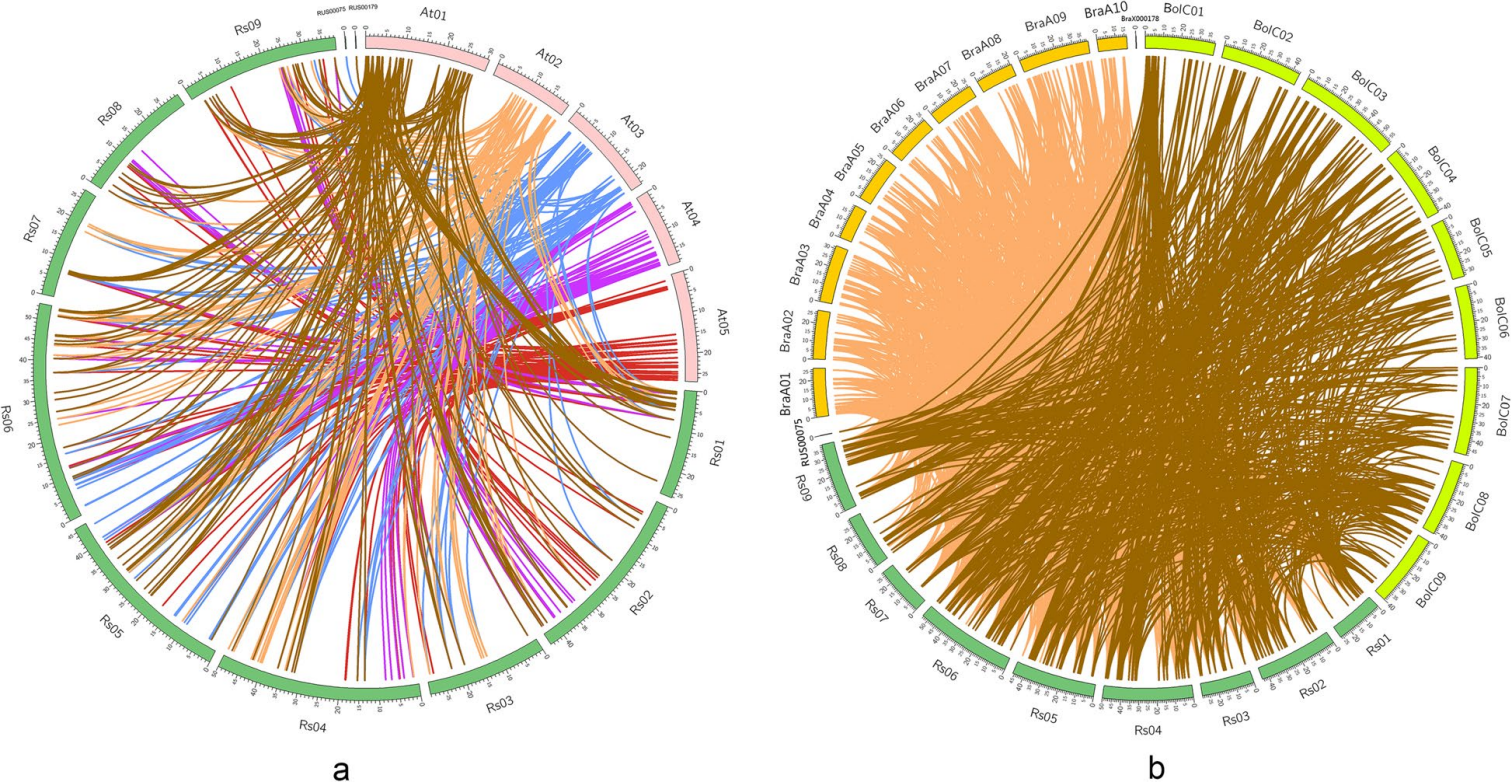
**a **The collinear analysis of *bHLH* genes between *Raphanus sativus* L. and *Arabidopsis thaliana*. The curves of brown, orange, blue, purple, and red link the *bHLH* genes on the At01, At02, At03, At04, and At05 chromosomes of *A. thaliana* and their syntenic orthologous genes in *Raphanus sativus* L., respectively. **b** The collinear analysis of *bHLH* genes between *Raphanus sativus* L. and *Brassica rapa*/*Brassica oleracea*. The curves of orange and brown link the syntenic orthologous *bHLH* genes between *Raphanus sativus* L. and *Brassica rapa*/*Brassica oleracea*, respectively

Syntenic genes among the related species are the orthologous genes located in the syntenic fragments, and they usually share the similar functions [10]. So, the syntenic gene analysis is a very important method to reveal the gene functions from the newly annotated genomes based on the gene functional information from the well-studied genomes (e.g., the model plant *Arabidopsis thaliana*). To understand the origins and functions of *RsbHLH* genes, a synteny relationship was analyzed between *Raphanus sativus* L. and *Arabidopsis thaliana*. The results indicated that there were 496 syntenic orthologous gene pairs between 176 *RsbHLH*s and 127 *AtbHLH*s (Fig. 6a). Among these gene pairs, 42 pairs of syntenic orthologous genes were one-to-one relationships, such as *RsbHLH100*-*AtbHLH26*, *RsbHLH170*-*AtbHLH110* and *RsbHLH55*-*AtbHLH11*. Some syntenic orthologous gene pairs with one *RsbHLH* corresponding to multiple *AtbHLHs* were also identified including *RsbHLH4*-*AtbHLH153*/*AtbHLH154*, *RsbHLH5*-*AtbHLH89*/*AtbHLH33*/*AtbHLH91*, *RsbHLH9*-*AtbHLH74*/*AtbHLH31*/*AtbHLH49*/*AtbHLH129*, etc. Correspondingly, there also existed syntenic orthologous gene pairs with one *AtbHLH* corresponding to multiple *RsbHLH*s, such as *AtbHLH160*-*RsbHLH21*/*RsbHLH185*, *AtbHLH155*-*RsbHLH75*/*RsbHLH151*, *AtbHLH106*-*RsbHLH48*/*RsbHLH77*. Additionally, other types of events were also discovered. For example, the gene pairs where two *RsbHLHs* correspond to the same two *AtbHLH*s were found, such as *RsbHLH2*-*AtbHLH26*/*AtbHLH132* and *RsbHLH158*-*AtbHLH26*/*AtbHLH132*, *RsbHLH3*-*AtbHLH7*/*AtbHLH59* and *RsbHLH43*-*AtbHLH7*/*AtbHLH59*, *RsbHLH4*-*AtbHLH153*/*AtbHLH154* and *RsbHLH166*-*AtbHLH153*/*AtbHLH154*. Syntenic orthologous gene pairs usually have similar functions, and these results could provide useful reference for further exploring the functions of *RsbHLH*. A series of syntenic events suggested that some *bHLH* genes generated before the divergence of *Raphanus sativus* L. and *Arabidopsis thaliana* lineages.

The Phylogenetic analyses of Brassicaceae species reveal that *R. sativus* belongs to the *Brassica rapa*/*Brassica oleracea* lineage not to *Brassica nigra* lineage, which supports that the *R. sativus* and *B. rapa*/*B. oleracea* have the closer relationship [11]. Additionally, the whole genome triplication (WGT) also exists in the *R. sativus* genome as is the case for the *B. rapa* and *B. oleracea* genomes [12]. *BHLH* genes have been confirmed in *B. rapa* and *B. oleracea*, with 251 *B. rapa bHLH*s and 268 *B. oleracea bHLH*s, respectively [13]. To understand the synteny relationships between *RsbHLH* genes and *Bra*/*BolbHLH* genes, the syntenic orthologous gene pairs were identified between *RsbHLH*s and *Bra*/*BolbHLH*s. There were 950 syntenic orthologous gene pairs between 174 *RsbHLH*s and 209 *BrabHLH*s (Fig. 6b). A total of 20 *RsbHLH*s only one syntenic orthologous *BrabHLH*s, such as *RsbHLH6*, *RsbHLH19*, *RsbHLH36*. The remaining *RsbHLH*s had at least two syntenic orthologous *BrabHLH*s. *RsbHLH*s with two syntenic *BrabHLH*s are the most, such as *RsbHLH61*- *BrabHLH080*/*BrabHLH218*. The number of *RsbHLH*s with four syntenic orthologous *BrabHLH*s ranked the second, such as *RsbHLH52*-*BrabHLH052*/*BrabHLH168*/*BrabHLH038*/*BrabHLH006*. Of course, there was also a case where multiple *RsbHLH*s correspond to one syntenic orthologous *BrabHLH*, such as *RsbHLH14*/*RsbHLH98*/*RsbHLH110*/*RsbHLH149*-*BrabHLH081*. There were 638 syntenic orthologous gene pairs between 162 *RsbHLH*s and 187 *BolbHLH*s (Fig. 6b). Like *BrabHLH*s, the syntenic relationship between *RsbHLH*s and *BolbHLH*s was divided into 1:1, 1:n, and n:1(n ≥ 2). A total of 24 *RsbHLH*s only one syntenic orthologous *BolbHLH*s, such as *RsbHLH37*, *RsbHLH121*, and *RsbHLH128*. *RsbHLH*s with two syntenic orthologous *BolbHLH*s had the highest number (such as *RsbHLH61*-*BolbHLH156*/*BolbHLH137*), followed by *RsbHLH*s with three syntenic orthologous *BolbHLH*s (*RsbHLH34*-*BolbHLH070*/*BolbHLH157*/*BolbHLH213*). Likewise, there were also multiple *RsbHLH*s corresponding to one syntenic orthologous *BolbHLH*, such as *RsbHLH8*/*RsbHLH40*/*RsbHLH175*/*RsbHLH193*- *BolbHLH080*. To sum up, although the number of *bHLH* genes in *B. oleracea* was slightly more than that of *B. rapa*, the syntenic orthologous *bHLH* pairs between *B. oleracea* and *R. sativus* was significantly less than that of *B. rapa* and *R. sativus*, which may be due to the closer relationship between *B. rapa* and *R. sativus*. This is supported by the genetic studies that the size and structural characteristics of the *R. sativus* genome are similar to those of *B. rapa* genome [14].

Genome duplication events can contribute to the expansion of gene family in plant kingdom. The results suggested that four types of duplication existed in the *RsbHLH* members, namely whole genome duplication (WGD) or segmental event, dispersed event, proximal event, and tandem event (Additional file 3). 162 (76%) *RsbHLH* genes were duplicated and retained from a WGD or segmental event, revealing that WGD or segmental duplication was the main driving force for the expansion of the radish *bHLH* gene family. Eight tandem events of 16 *RsbHLH* genes were identified and located on four chromosomes. Among these events, two events (*RsbHLH10* and *RsbHLH11*, *RsbHLH96* and RsbHL*H97*) were located on chromosome R1 and R5, respectively. Three events (*RsbHLH57* and *RsbHLH58*, *RsbHLH59* and *RsbHLH60*, and *RsbHLH69* and *RsbHLH70*) took place within the same chromosome R4, and the remaining three events (*RsbHLH133* and *RsbHLH134*, *RsbHLH136* and *RsbHLH137*, and *RsbHLH144* and Rsb*HLH145*) also took place within the same chromosome R6. Finally, the dN / dS for the 48 paralogous gene pairs were calculated to confirm the selection pressure (Additional file 4). All of the *RsbHLH* paralogous gene pairs had a dN / dS < 1(dN means non-synonymous substitution ratio; dS means synonymous substitution ratio), implying that these *RsbHLH* genes had experienced strong purifying selective pressure.

### Promoter cis-element analysis

**Fig. 7:**
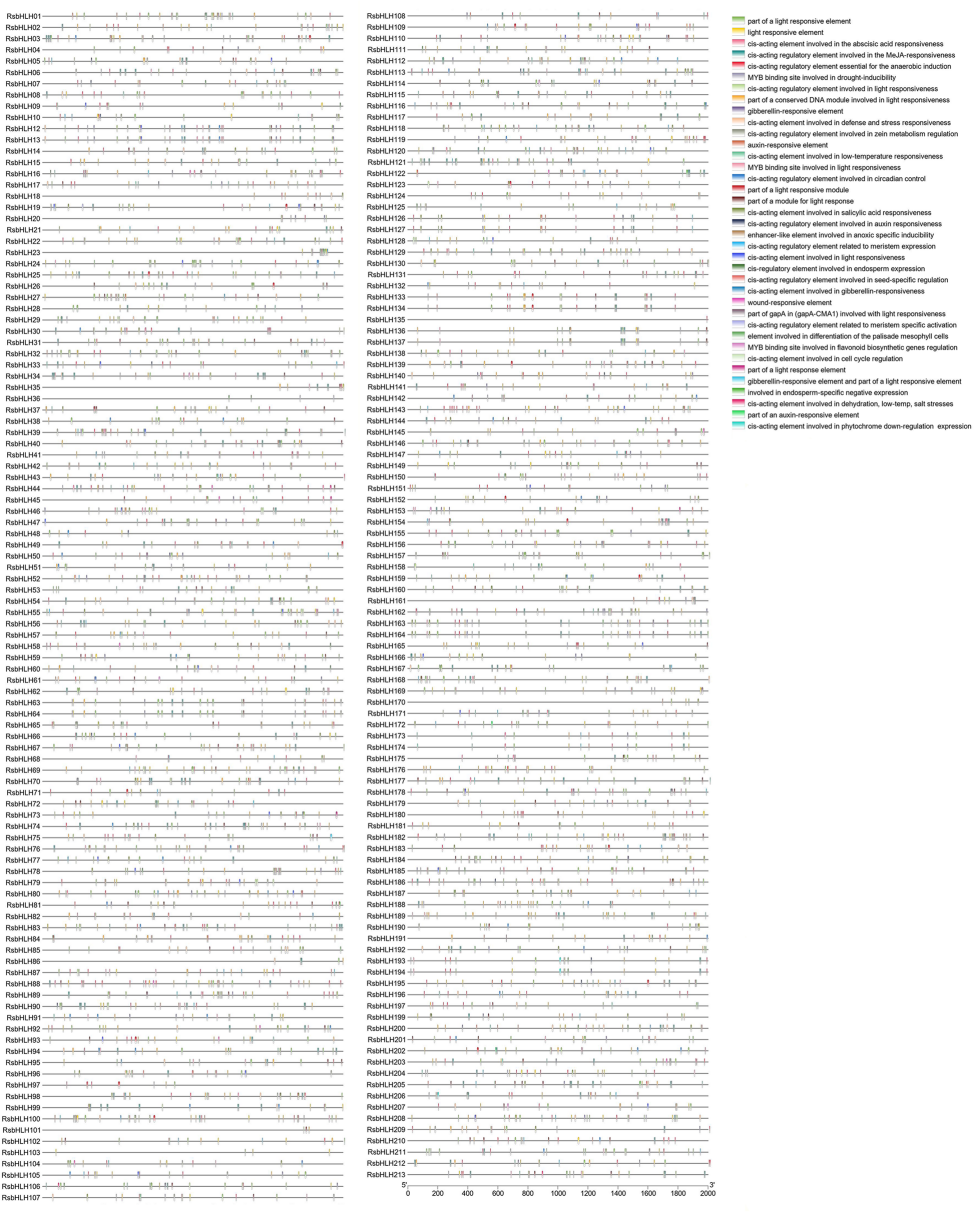
Cis-regulatory elements in the promoter regions of the *RsbHLH* genes. Different cis-regulatory elements are represented with different colored boxes, which are placed at the top on the right. The element size is estimated by the scale at the bottom

*BHLH* genes can take part in the regulation of plant growth and development, and response to various abiotic stresses. To further investigate the potential biological functions of *RsbHLH* genes, the cis-acting regulatory elements in the promoter regions of *RsbHLH* genes were analyzed using PLACE tool. As shown in Fig. 7 and Additional file 5, three main categories were identified in the cis-acting regulatory elements of *RsbHLH* genes. Category one was associated with plant growth and development, such as flavonoid biosynthesis, phytochrome expression, and circadian control. The motifs contained in this category were MBSI, circadian, CAT-box, RY-element, etc. Light responsive element was present in the promoter regions of 208 *RsbHLH* genes, indicating that the expression of *RsbHLH* genes maybe controlled by light. The genes involved in chlorophyll metabolism are regulated by light [15]. This may mean that *RsbHLH* genes had a certain relationship with chlorophyll metabolism. Category two was related to phytohormones, such as gibberellin, methyl jasmonate, and abscisic acid. The motifs included in this category were CGTCA-motif, ABRE, TCA-element, P-box, etc. Category three was involved in abiotic stresses, such as low-temperature responsiveness, light responsiveness, and drought-inducibility. The motifs included in this category were LTR, G-box, MBS, WUN-motif, etc.

### Analysis of GO enrichment and expression

**Fig. 8:**
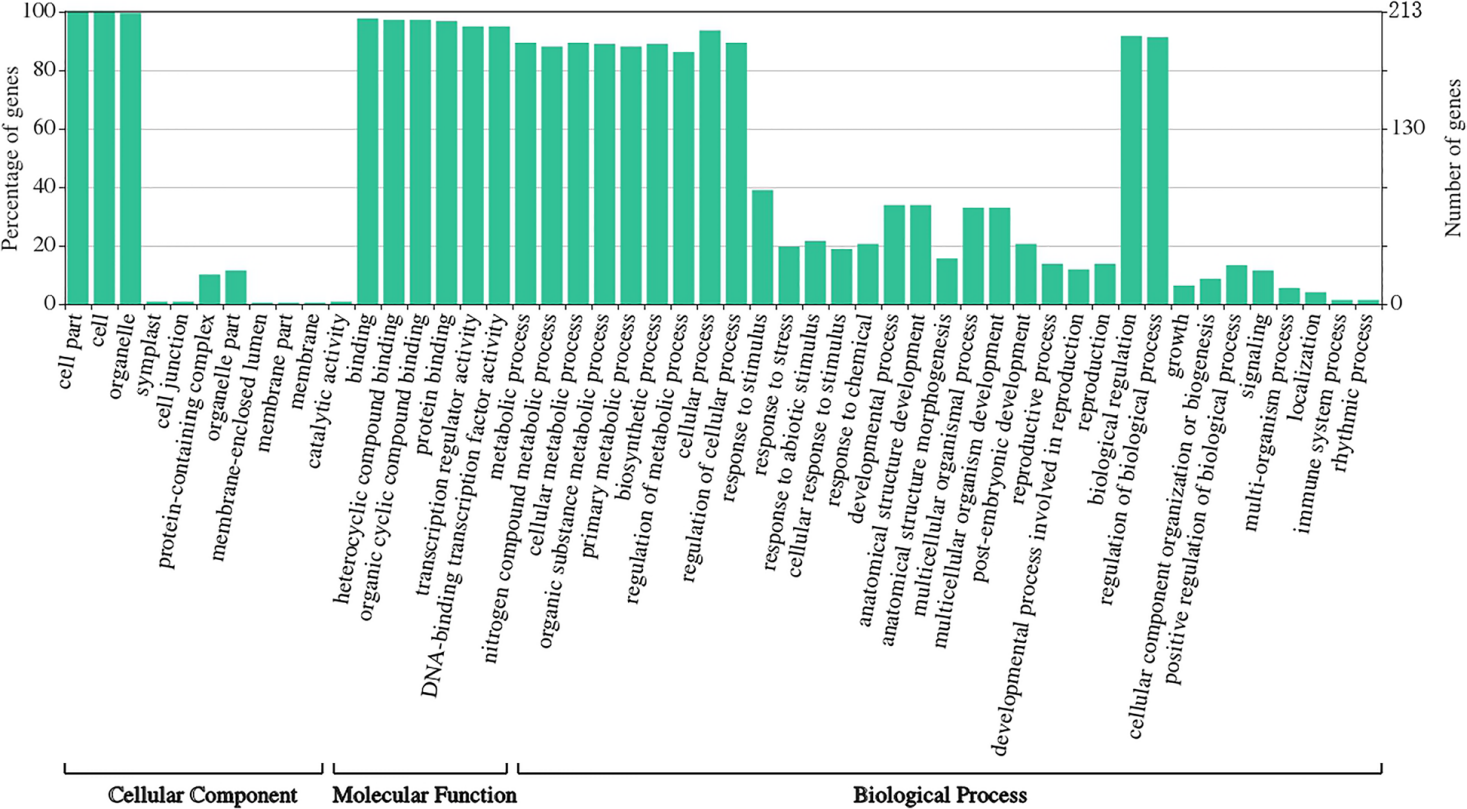
The GO annotation of *RsbHLH* genes. All annotated GO terms include cellular component, molecular function and biological process. The y axis indicates the number of genes

To predict the potential biological functions, the GO enrichment analysis was performed by WEGO to show three aspects of functional classifications, namely, cellular component, molecular function, and biological process (Fig. 8). Among the 213 *RsbHLH* genes, 207 *RsbHLH* genes were enriched in the biological process, and most of these *RsbHLH*s mainly participated in “metabolic process” (such as “primary metabolic process” and “biosynthetic process”), and “biological regulation” (such as “regulation of metabolic process” and “regulation of cellular process”). Additionally, 85 *RsbHLH*s, 74 *RsbHLH*s and 72 *RsbHLH*s were involved in “response to stimulus”, “developmental process”, and “multicellular organismal process”, respectively. The “response to stimulus” was mainly associated with “response to abiotic stimulus” (cold, salt, light, etc.) and “response to chemical” (gibberellin, abscisic acid, jasmonic acid, etc.), which was consistent with the previous promoter element analysis. The “developmental process” involved mainly in “anatomical structure development” (fruit development, flower development, carpel development, etc.). The “multicellular organismal process” contained mainly photomorphogenesis, guard cell differentiation, root hair initiation and so on. In summary, *RsbHLH*s could play an important role in the growth and development of radish.

**Fig. 9:**
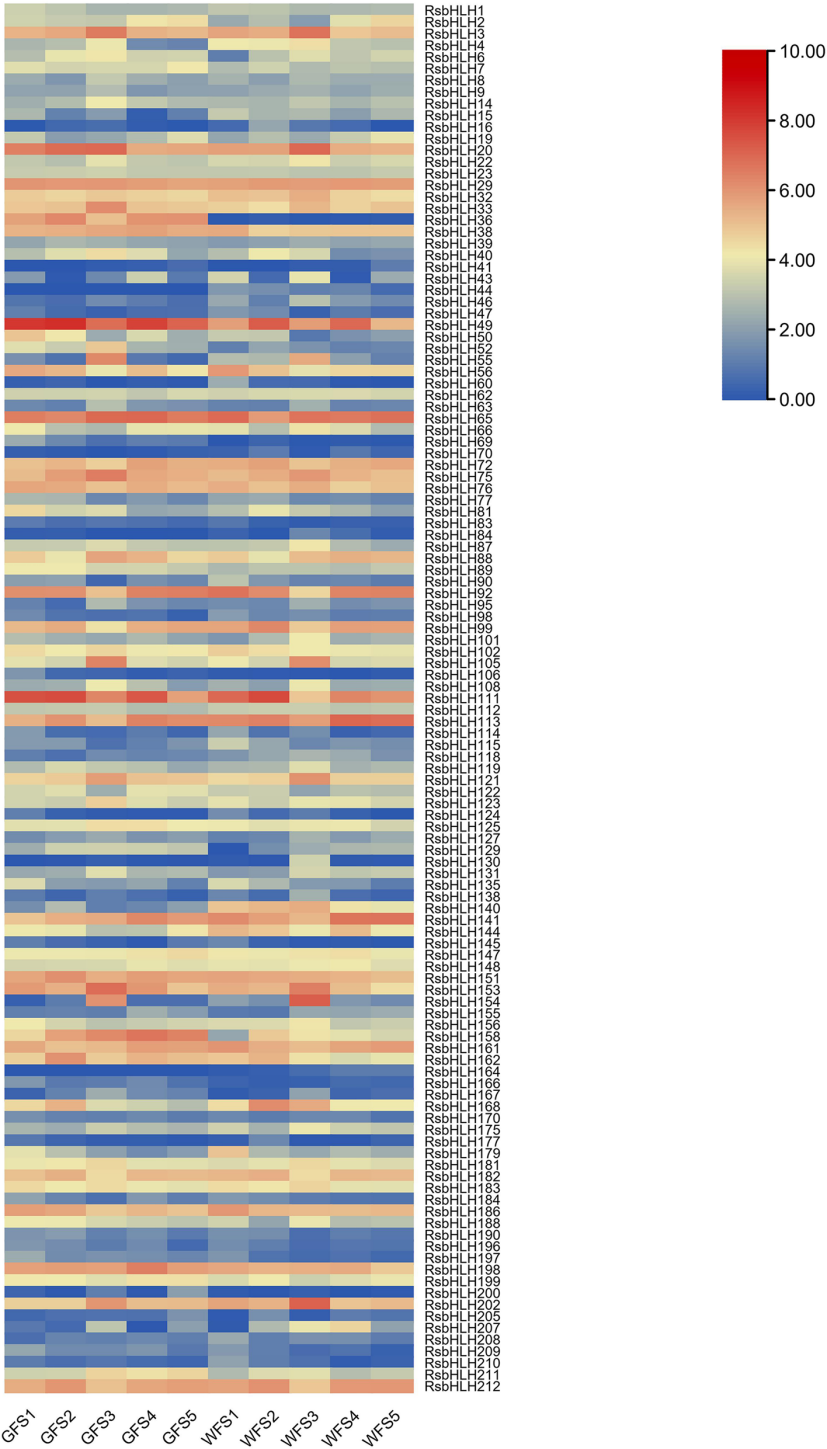
Expression patterns of *RsbHLH* genes at five development stages of GF and WF. GF indicates the green-fleshed radish ‘Cuishuai’; WF indicates the white-fleshed radish ‘Zhedachang’. The colour scale is shown at the right top. Higher expression level is in red, while lower expression level is in blue

A comparative RNA-seq analysis between the green-fleshed radish (GF) and white-fleshed radish (WF) was made to study the expression patterns of 213 *RsbHLH*s at five growth stages. The *RsbHLH* genes with FPKM < 1 at all five stages in GF and WF were regarded as unexpressed genes, so only 119 *RsbHLH* genes were carried on expression analysis. The expression patterns of these 119*RsbHLHs* among five stages varied greatly (Fig. 9). For example, whether GF or WF, some *RsbHLH*s were stably expressed at five stages, such as *RsbHLH29*. Additionally, whether GF or WF, *RsbHLH105* and *RsbHLH154* were only highly expressed at the stage 3. For *RsbHLH36*, it had always been highly expressed at five stages in GF, while always lowly expressed at five stages in WF. Reversely, *RsbHLH140* had always been highly expressed at five stages in WF, while always lowly expressed at five stages in GF. For *RsbHLH130*, it only had a relatively high expression at the stage 3 of WF. The various expression patterns of *RsbHLH*s among five stages suggested that the *bHLH* members may play a vital role in the entire growth and development of radish taproot. The differentially expressed gene (DEG) analysis revealed that there were 19, 16, 22, 18, 13 differentially expressed *RsbHLH*s between GF and WF at stage 1 to stage 5, respectively (Additional file 6). Four *RsbHLH*s (*RsbHLH36*, *RsbHLH44*, *RsbHLH69*, and *RsbHLH140*) were differentially expressed genes shared by the five stages. The differential expression of *RsbHLH*s may lead to the differences in traits between green-fleshed radish and white-fleshed radish.

To demonstrate the accuracy and reproducibility of the transcriptome data, fifteen *RsbHLH* genes were selected to analyze the transcript abundance by qRT-PCR. The results showed that the expression trends of these fifteen genes detected by qRT-PCR in the fifth stage were in line with the RNA-Seq results (Additional files 7 and  8). Therefore, the reliability of the transcriptome data was confirmed.

### Candidate *bHLH*s involved in chlorophyll metabolism in *Raphanus sativus* L

**Fig. 10:**
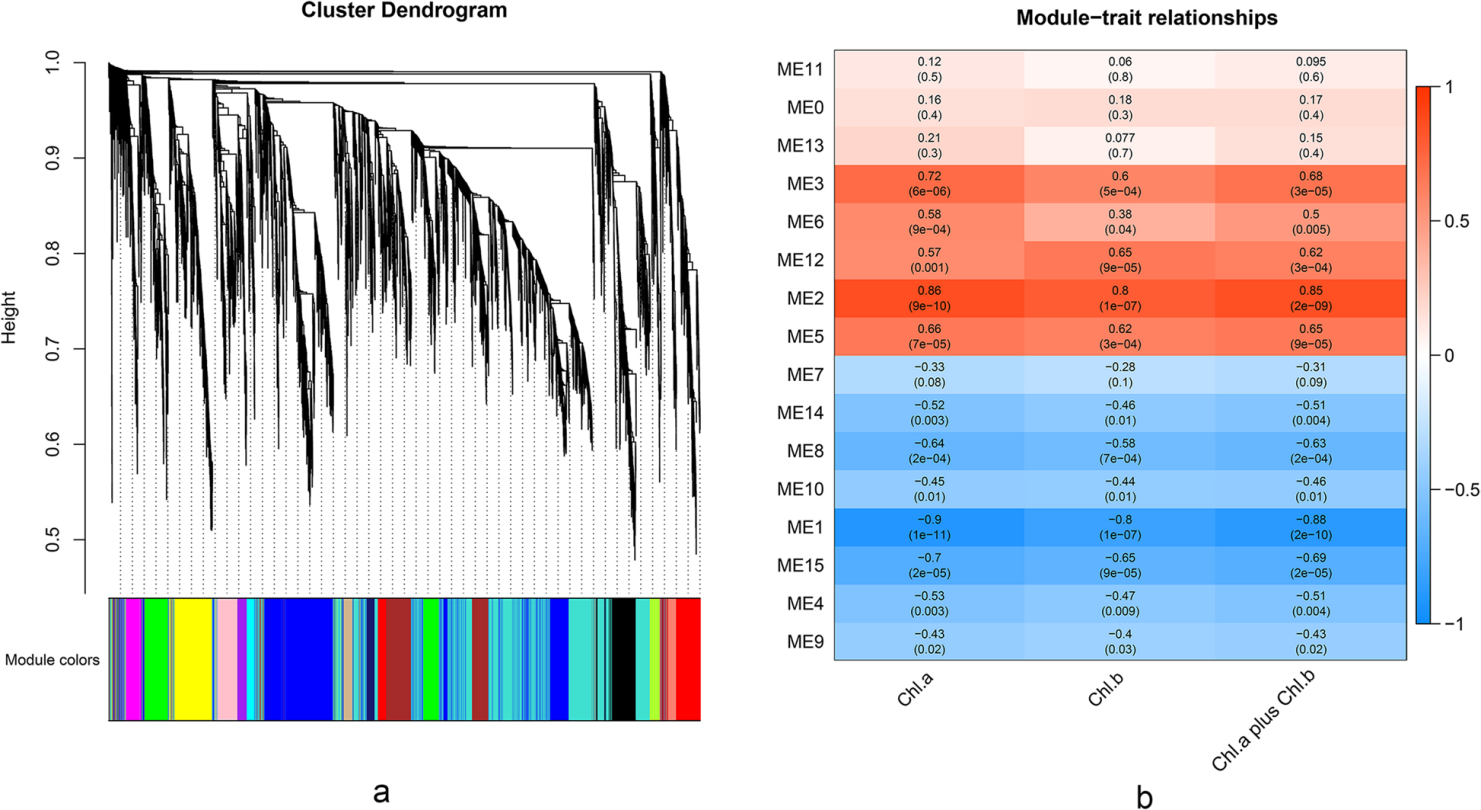
**a** Hierarchical cluster tree revealing gene co-expression modules identified by WGCNA. The branches contain 15 modules labeled in different colors. Except the gray module, the modules are named ME1 to ME15. **b** Module-trait associations. Columns correspond to chlorophyll content, and rows correspond to the characteristic genes of the modules. The correlation between two is shown in cell by Pearson correlation coefficient, and p-value is in parentheses. Cell color ranges from red (high positive correlation) to blue (high negative correlation)

The weighted gene co-expression network analysis (WGCNA) was used to analyze the connection between genes and physiological traits, discovering the vital genes associated with physiological traits. There were 8666 DEGs between GF and WF, including 46 *bHLH* genes, and these DEGs were performed by WGCNA. The expression profiles of the 8666 genes were grouped into 16 modules (MEs), displaying 15 different co-expression networks (ME1-ME15) and the outliers that did not belong to any cluster (ME0). As Fig. 10a shown, different colors represented different modules, with the module size ranging from 27 to 2510. To confirm modules that were significantly associated with chlorophyll content, the module-trait correlation relationships were constructed (Fig. 10b). Chlorophyll a content was significantly negatively correlated with the turquoise module (-0.9) and midnightblue module (-0.7), and significantly positively associated with the blue module (0.86) and brown module (0.72). Chlorophyll b was significantly positively correlated with the blue module (0.8), and significantly negatively correlated with the turquoise module (-0.8). These four modules contained 23 *bHLH* genes, which were used to screen out hub genes.

Gene with high within-module connectivity was considered as a hub gene in a module [16]. Hub genes in modules could be more important than the other genes in the co-expression network, and they were considered as the representatives of the modules. Based on the high connectivity, four *bHLH* genes were regarded as hub genes: *RsbHLH140* (turquoise), *RsbHLH52* (blue), and *RsbHLH36* and *RsbHLH49* (brown). DEGs co-expressed with these four *bHLH* genes were screened out for further analysis.

**Fig. 11:**
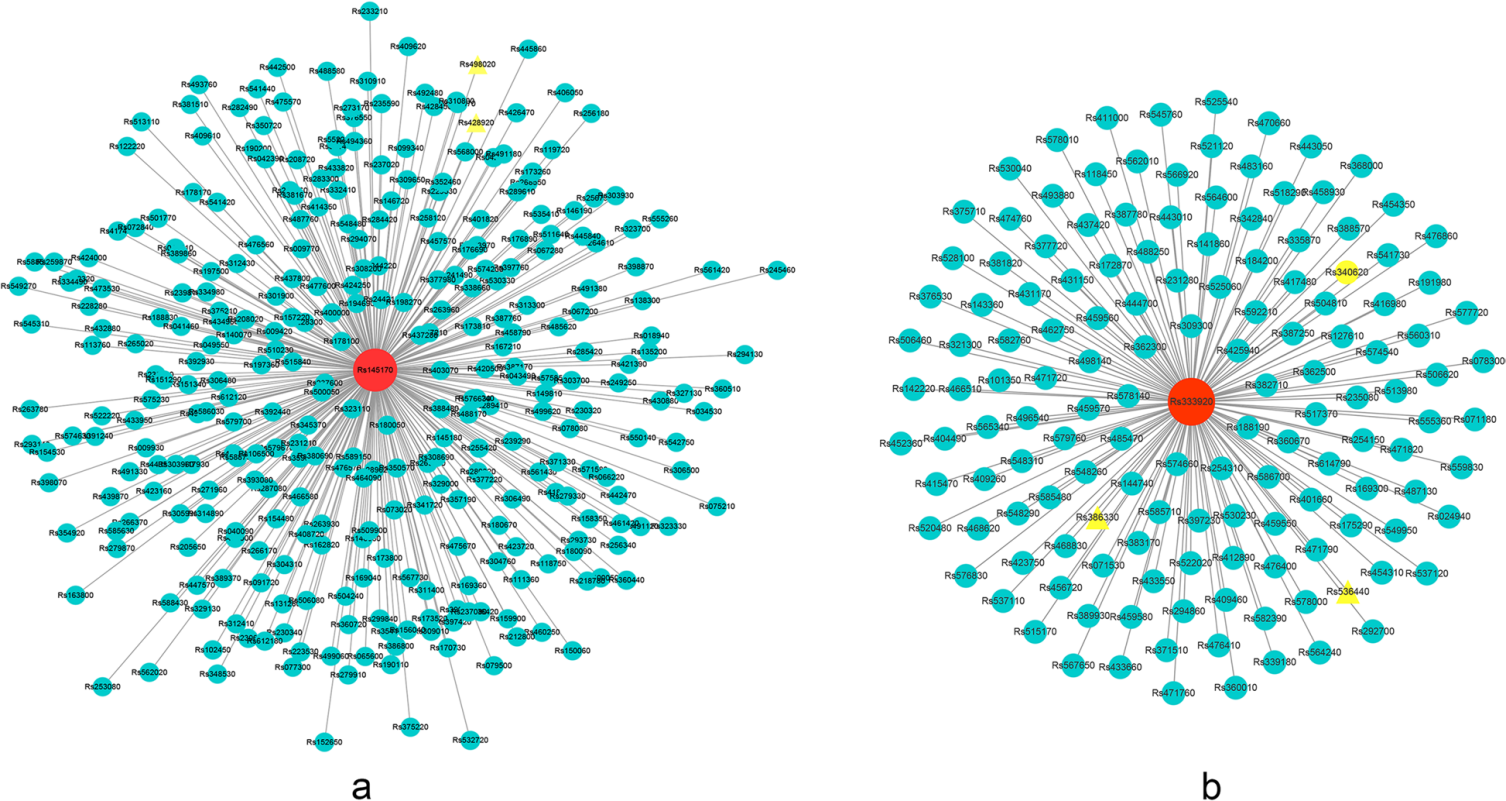
DEGs co-expressed with *RsbHLH52* in the blue module (**a**) and *RsbHLH140* in the turquoise module (**b**)

Two DEG genes (*Rs498020* and *Rs428920*) co-expressed with *RsbHLH52* and three DEG genes (*Rs536440*, *Rs386330*, and *Rs340620*) co-expressed with *RsbHLH140* were found to be involved in the chlorophyll metabolic pathway [1], and these five genes were shown in Fig. 11. Furthermore, as shown in Fig. 10b, *RsbHLH140* was negatively correlated with chlorophyll content, while *RsbHLH36*, *RsbHLH49*, and *RsbHLH52* were positively correlated with chlorophyll content. Thus, it was concluded that *RsbHLH140* could negatively regulated the process of chlorophyll metabolism, and *RsbHLH36*, *RsbHLH49*, and *RsbHLH52* positively controlled the process of chlorophyll metabolism, which was consistent with the analysis results of the gene expression pattern (Fig. 9). To sum up, these four *bHLH* genes may be involved in the metabolic process of chlorophyll, and then associated with GF photosynthesis.

## Discussion

### The whole genome duplication greatly facilitates the expansion of the *bHLH* gene family

The whole genome duplication (WGD) events have occurred throughout the process of plant evolution, which was a driving force for the expansion of gene family [17]. In radish, some gene families expanded also mainly through the whole genome duplication to generate the large number of members, such as *MYB* gene family, *HSF* gene family and *CPA* gene family [18–20]. In this study, 213 *bHLH* genes were identified from the radish genome, showing that the *bHLH* gene family in radish had been expanded in comparison with that in *Arabidopsis*. The results showed that 162 (76%) *RsbHLH* genes were duplicated and retained in the WGD event, implying that WGD event greatly promoted the amplification of the *RsbHLH* gene family. This result was consistent with the results of the *bHLH* gene family analysis in other species, such as *Nelumbo nucifera*, *Fagopyrum tataricum*, and *Xanthoceras sorbifolia Bunge* [21–23].

Gene duplication promotes the formation of the paralogous gene pairs. A total of 48 paralogous gene pairs were identified in our results, including 8 paralogous gene pairs from the tandem duplication and 36 paralogous gene pairs from the WGD duplication. Among these paralogous gene pairs, 16 paralogous gene pairs had the different gene structure, and exon gain/loss was observed. For instance, *RsbHLH17* contained four exons, while its paralogous *RsbHLH170* had seven exons (Fig. 2), indicating a gain of three exons occurred during evolution. *RsbHLH138* contained six exons, while its paralogous *RsbHLH193* had four exons, indicating a loss of two exons occurred during evolution. A similar pattern was also reported in *bHLH* gene families of the *Ginkgo biloba* and *Xanthoceras sorbifolia Bunge* [23, 24]. These gain/losses could be due to the results of chromosomal rearrangements and fusions, and may potentially give rise to the functional diversification of the gene families [25]. Additionally, 12 paralogous gene pairs had the discrepant motifs, in which four gene pairs had the same gene structures, such as *RsbHLH10*-*RsbHLH11* and *RsbHLH74*-*RsbHLH152*. Differences in gene sequences may lead to changes in protein domains although they had the same gene structures. These proteins may have gone through the wide domain shuffling during the WGD [26]. We also found that despite some paralogous gene pairs had the different gene structures, they had the same motifs, such as *RsbHLH52*-*RsbHLH161* and *RsbHLH93*-*RsbHLH124*, revealing that the sequences of protein motifs were conserved in the process of evolution.

The duplicated genes could acquire the new functions or segment the original functions to improve the adaptability of environments [27]. There are four fates for duplicated genes: (1) duplicated genes retain original functions; (2) a copy of the duplicated genes is silenced; (3) a copy of the duplicated genes retains the original function, while another copy obtains the new function, called neofunctionalization; (4) Two copies segment the original functions and obtain different functions, called subfunctionalization [28, 29]. The functional divergence of duplicated genes could cause the alteration in the expression pattern. Herein, some paralogous gene pairs showed the different expression patterns. For example, *RsbHLH2* was expressed at five stages of the GF and WF, while its paralogous *RsbHLH100* was not expressed at five stages of the GF and WF. *RsbHLH92* had the lowest expression level at the third stage of the GF and WF, while its paralogous *RsbHLH123* had the highest expression level at the third stage of the GF and WF. In a word, the distinct expression patterns of the paralogous *bHLH* gene pairs may lead to the formation of the unique traits and improving the adaptability to the environment in radish.

### *BHLH* genes may have important functions in the photosynthesis in green-fleshed radish

Transcription factors (TFs) are activated and bind to the promoter of the crucial genes involved in various biological pathways, and regulate the growth and development of plants. As one of the most important biological pathways for plants, photosynthesis is regulated by a variety of transcription factors [30–32]. The *bHLH* gene family is the second largest TF family, playing an important role in the regulation of photosynthesis in plants [33].

In addition to leaves, photosynthesis also appears in many non-foliar organs. The results of chlorophyll fluorescence show that photosynthesis can occur in the green-fleshed radish rich in chlorophyll [1]. In our study, four *bHLH* genes (*RsbHLH36*, *RsbHLH49*, *RsbHLH52*, and *RsbHLH140*) may participate in controlling the photosynthesis process by influencing the changes of chlorophyll content. In the *bHLH* gene family, a few members are generally considered as negative regulators of photosynthesis. For example, phytochrome interacting factor1 (*PIF1*) and phytochrome interacting factor4 (*PIF4*), they negatively regulate the chlorophyll biosynthesis or bring about the chlorophyll degradation to lower the chlorophyll content, hindering the progress of photosynthesis [6, 9]. According to the WGCNA analysis, *RsbHLH140* was significantly negatively correlated with the chlorophyll content. In addition, compared with GF, *RsbHLH140* had the higher expression level in WF (Fig. 9). Three DEGs (*Rs536440*, *Rs386330,* and *Rs340620*) co-expressed with *RsbHLH140* were involved in chlorophyll biosynthesis pathway, and they were expressed in GF, while hardly expressed in WF [1], showing that the higher expression of *RsbHLH140* may suppress the expression of these three genes in WF. The *A. thaliana* orthologous genes of these three genes (*AT3G56940*, *AT1G74470*, and *AT3G51820*) are annotated in the TAIR database and participate in the chlorophyll biosynthetic process [34]. So, *RsbHLH140* may act as a negative regulator of photosynthesis by suppress the chlorophyll biosynthesis. It is reported that knocking out of the negatively regulated *bHLH* gene can enhance the photosynthesis. Chen et al. [35] find that knocking out *NRP1* gene, as a *bHLH* gene, gives rise to greater photosynthesis and increased biomass in rice.

Additionally, a few *bHLH* genes are regarded as positive regulators of photosynthesis. In contrast to *PIF1*, *PIF3* has been proved as a positive regulator of photosynthesis. *PIF3* contributes to the chlorophyll accumulation and acts positively in chloroplast development [36]. Overexpression of *PebHLH35* from *Populus euphratica* results in a higher chlorophyll content and enhance the photosynthetic rate [7]. In our results, *RsbHLH36*, *RsbHLH49*, *RsbHLH52* were significantly positively correlated with the chlorophyll content, and the expression levels of these three genes in GF were higher than that in WF at five stages. In particular, *RsbHLH36* was significantly highly expressed in the GF compared to WF. They may act as a positive regulator of photosynthesis by contributing to the chlorophyll accumulation.

To sum up, *RsbHLH* genes may function in the process of photosynthesis by altering the content of chlorophyll in the flesh of GF taproot.

## Conclusion

The *bHLH* gene family were comprehensively and systematically characterized in this study. A total of 213 *RsbHLH* genes were genome-widely identified in the genome of *Raphanus sativus* L. The gene structure analysis showed that some *RsbHLH* genes were intron-less. The gene duplication analysis suggested that WGD event played a major role in the expansion of *RsbHLH* gene family. The analysis of promoter cis-element, expression pattern and WGCNA showed that *RsbHLH* genes might be involved in the process of chlorophyll metabolism. In addition, four *RsbHLH* genes were regarded as hub genes, they could act as the negative or positive regulator of photosynthesis by controlling the chlorophyll content. This study laid a foundation for further exploring and understanding the molecular mechanisms of photosynthesis in the green-fleshed radish.

## Materials and methods

### Plant materials

The green-fleshed radish ‘Cuishuai’ (GF) and white-fleshed radish ‘Zhedachang’ (WF) were planted in the outdoor big field in Weifang (36.62 degrees north latitude and 119.10 degrees east longitude), Shandong, China under natural conditions on August 31(autumn). Taproot flesh tissues of GF and WF were collected every seven days from September 25 to October 23 (a total of five developmental stages, S1-S5). There were three biological replicates in each stage for GF and WF. The flesh tissues were frozen in liquid nitrogen as quickly as possible, and then stored at − 80 °C for later RNA-seq analysis.

### Identification of *RsbHLH* genes

In *Arabidopsis thaliana* database (TAIR, http://www.arabidopsis.org), a total of 158 *bHLH* genes were found, and the information of 158 *AtbHLH* genes was added to Additional file 1. The protein sequences of *Raphanus sativus* L. were retrieved from the RadishGD (Radish Genome DataBase, http://radish-genome.org/), and the version was Rs1.0. The sequences of 158 *Arabidopsis* bHLH proteins (AtbHLHs) were downloaded from the TAIR database (http://www.arabidopsis.org). The Hidden Markov Model (HMM) profile of the bHLH domain (PF00011) was obtained from the Pfam database (http://pfam.xfam.org/). AtbHLHs were used as queries for a BLASTP analysis against the protein sequences of *Raphanus sativus* L. with a cut-off E-value ≤ 1 e ^−5^. Then, the hmmsearch of the HMMER (version 3.0) software was applied to screen and identify *RsbHLH* genes based on the HMM profile (PF00011), with a cut-off E-value ≤ 1 e ^−5^. Finally, these two searches were combined to make a non-redundant *RsbHLH* candidate list. The *RsbHLH* candidates were analyzed to confirm the existence of the conserved bHLH domain by NCBI Conserved Domain Search (https://www.ncbi.nlm.nih.gov/Structure/cdd/wrpsb.cgi), InterProScan (http://www.ebi.ac.uk/interpro/sequencesearch) and SMART (http://smart.embl-heidelberg.de). The *RsbHLH* candidates with complete bHLH domains were retained and renamed based on their position information on *Raphanus sativus* L. chromosomes.

### Gene location, structure, duplication pattern and syntenic analysis

To localize the *RsbHLH* genes on chromosomes, the position information of *RsbHLH* genes were collected from GFF files in *Raphanus sativus* L. Genome DataBase. The location map of *RsbHLH* genes on 9 *Raphanus sativus* chromosomes was conducted using the MapChart software [37]. The structures of the *RsbHLH* genes were displayed to illustrate the exon–intron composition using the Gene Structure Display Server (GSDS, http://gsds.cbi.pku.edu.cn). The duplication types of *RsbHLH* genes were examined using MCScanX [38]. Syntenic gene pairs between *Raphanus sativus* L. and *Arabidopsis thaliana* were searched by MCScanX, and the syntenic relationship pairs were shown by Circos [39].

The non-synonymous (dN) / synonymous (dS) substitution values between the *RsbHLH* paralogous genes were analyzed to identify the mode of selection. The protein sequences of each *RsbHLH* paralogous pair were aligned using MAFFT. Next, the alignment results and the corresponding DNA sequences were imported into PAL2NAL tool to convert into the relevant codon alignments. Finally, the codon alignment results were carried on the calculation of non-synonymous (dN) and synonymous (dS) substitution rates by the codeml program in PAML [40].

### Analysis of conserved motif, phylogenetic relationship and classification of the RsbHLH proteins

To find out the conserved motifs, the sequences of all RsbHLH proteins were imported into MEME (https://meme-suite.org/meme/tools/meme). The multiple sequence alignment was performed based on the sequences of RsbHLH proteins and AtbHLH proteins using the MAFFT software, the alignment result was used to construct the phylogenetic tree with Neighbor-Joining (NJ) method generated by MEGA7.0 software (poisson model, pairwise deletion option and 1000 bootstrap replicates). Subfamily grouping of the RsbHLH proteins was constructed on the basis of the classification scheme of the AtbHLH proteins.

### Search of cis-elements in the *RsbHLH* gene promoter regions

The upstream 2000 bp genomic sequences of *RsbHLH* genes relative to the translation start codon were extracted from *Raphanus sativus* L. genome. These 2000 bp regions were regarded as the promoter sequences of *RsbHLH* genes. The cis-regulatory elements of *RsbHLH* genes were screened from these promoter regions using online tool PLACE (https://www.dna.affrc.go.jp/PLACE/?action=newplace).

### RNA-seq and qRT-PCR analysis of *RsbHLH* genes

Total RNA was extracted from the flesh tissues using Trizol reagent (Promega, Madison, WI, USA). The purity, concentration, and integrity of RNA were detected by the NanoPhotometer® spectrophotometer (IMPLEN, CA, USA), the Qubit® 2.0 Flurometer (Life Technologies, CA, USA), and the Bioanalyzer 2100 system (Agilent Technologies, CA, USA), respectively. After the RNA quality assessment, 30 sequencing libraries were generated using the NEBNext® Ultra™ Directional RNA Library Prep Kit according to the Illumina manufacturer's protocols (NEB, USA). The quality of libraries was checked by the Agilent Bioanalyzer 2100 system. Finally, 150 bp paired-end sequencing was performed on an Illumina Hiseqxten platform. After screening, the high-quality clean reads were aligned to the reference *Raphanus sativus* L. genome using the HISAT2 software [41]. The mapped clean reads were calculated to obtain the read count for each gene according to the mapping results by the featureCounts software [42]. The expression level of each gene was estimated using the fragments per kilobase of exon per million mapped reads (FPKM) value, which was calculated using the countToFPKM package (https://github.com/AAlhendi1707/countToFPKM). The formula is as follows: $\text{FPKM} = \frac{{10}^{6}{\text{C}}}{\text{NL/}{10}^{3}}$, where C is the number of fragments that specially mapped to the gene, N is the total number of fragments that specially mapped to the reference genome, and L is the number of bases in the coding region of the gene. Genes were considered to be differentially expressed genes (DEGs) between GF and WF with FDR value ≤ 0.05, Padj value < 0.01and |log_2_ FC|≥ 1.5 based on the DEGSeq R package [43]. The expression patterns of *RsbHLH* genes in the five developmental stages were presented by the TBtools software [44].

Fifteen *bHLH* genes were randomly chosen to be verified by qRT-PCR. The specific primers of fifteen genes were designed by the Primer5 software, and the primer sequences were listed in Additional file 9. QRT-PCR was performed using the Bio-Rad Real-Time PCR platform with quant one step qRT-PCR Kit (Tian gen). *Actin* gene was used as the internal control to standardize the results, and 2^−ΔΔCT^ method was applied to calculate the relative expression level [45]. The all reactions were carried out with the following conditions: 95 °C for 15 min and 40 cycles of 95 °C for 10 s, 60 °C for 30 s. After each run, a melting curve was generated to ensure the product specificity and to check for the presence of primer dimers.

### The weighted gene co-expression network analysis

The co-expression network of DEGs was constructed using WGCNA package in Rstudio [46]. The PickSoftThreshhold function was used to confirm a soft threshold (power) value according to the approximate Scale-free Topology Criterion. The soft threshold was 10 to establish the co-expression network on the basis of the adjacency matrix. The automatic network construction function blockwiseModules was applied to obtain weighted co-expression clusters, called modules, with the following operating parameters: power = 10, TOMType = unsigned, minModuleSize = 30, reassignThreshold = 0, minKMEtoStay = 0.3, mergeCutHeight = 0.25. The data on chlorophyll content was derived from our previous research results [1].

## Supplementary Information

**Additional  file 1: Table S1.** The information of the identified bHLH genes in Raphanus sativus L. and Arabidopsis thaliana

**Additional  file 2: FigureS1. **Sequencelogos of 19 conserved motifs. The height of a letter indicates its relativefrequency at the given position

**Additional  file 3: Table S2.** The duplication type of bHLH genes in Raphanus sativus L. WGD means whole genome duplication

**Additional  file 4: Table S3.** Non-synonymous and synonymous substitution rates of the 48 paralogous bHLH gene pairs. dN means non-synonymous substitution ratio; dS means synonymous substitution ratio

**Additional  file 5: Table S4.** The list of cis-elements in RsbHLH gene promoters

**Additional  file 6: Table S5.** The differentially expressed RsbHLH genes between GF and WF at stage 1 to stage 5

**Additional  file 7: Figure S2. **The expression levels of the fifteen *RsbHLHs* at the fifth stage of GF and WF by qRT-PCR andRNA-seq. qRT-PCR were normalized to the expression of *Actin*

**Additional  file 8: Table S6.** The relative expression data of the fifteen RsbHLHs at the fifth stage of GF and WF by qRT-PCR.

**Additional  file 9: Table S7.** Primer sequences of the selected fifteen bHLH genes for qRT-PCR in Raphanus sativus L

## Data Availability

The raw data of RNA-seq had been deposited to Sequence Read Archive (SRA) in NCBI (accession number: PRJNA684971).

## Abbreviations

BHLH: Basic helix-loop-helix
GSDS: Gene Structure Display Server
MEME: Multiple Em for Motif Elicitation
HMM: Hidden Markov Model
dS: Synonymous
dN: Non-synonymous
NJ: Neighbor-Joining
FPKM: Fragments per kilobase of exon per million mapped reads
WGD: Whole genome duplication
DEG: Differentially expressed gene
qRT-PCR: Quantitative Real-Time Polymerase Chain Reaction
WGCNA: Weighted gene co-expression network analysis
