# Supplementary material for: Genome-wide identification and characterization of the bHLH gene family and analysis of their potential relevance to chlorophyll metabolism in Raphanus sativus L
Source: BMC Genomics. 2022 Aug 1;23:548. doi: 10.1186/s12864-022-08782-4 (PMC9344636; doi:10.1186/s12864-022-08782-4)

**Figure S1** Sequence logos of 19 conserved motifs. The height of a letter indicates its relative frequency at the given position.


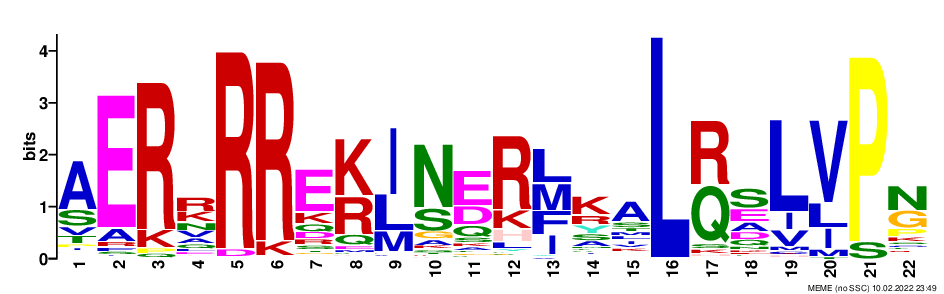


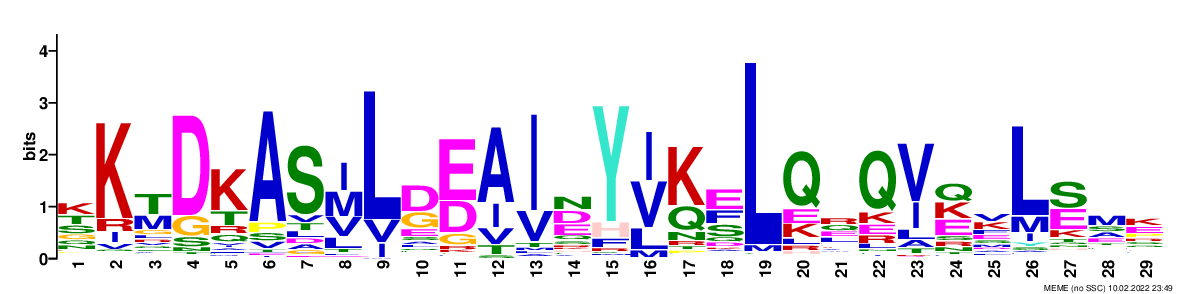


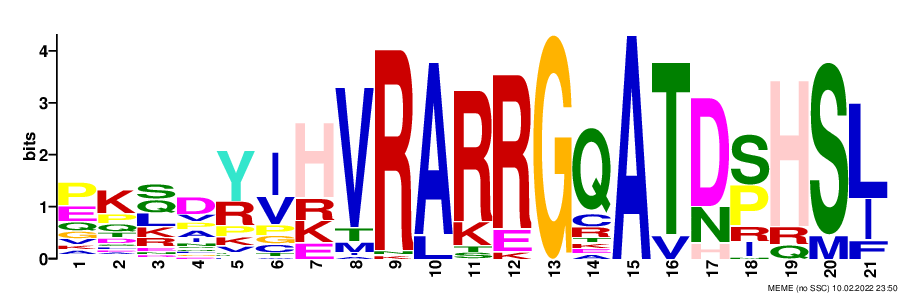


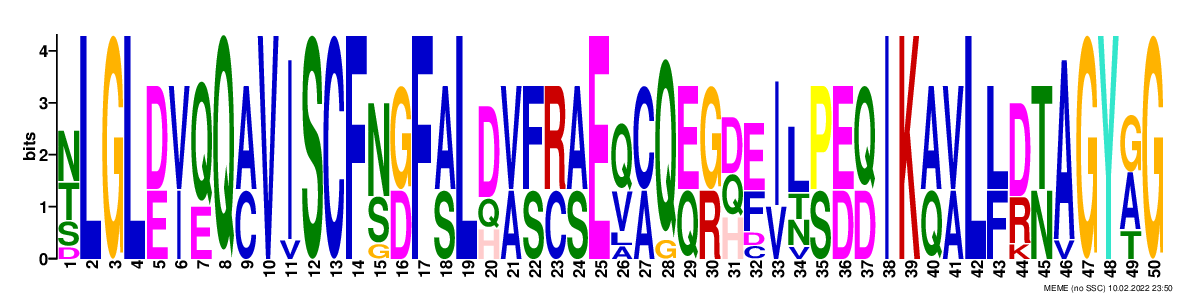


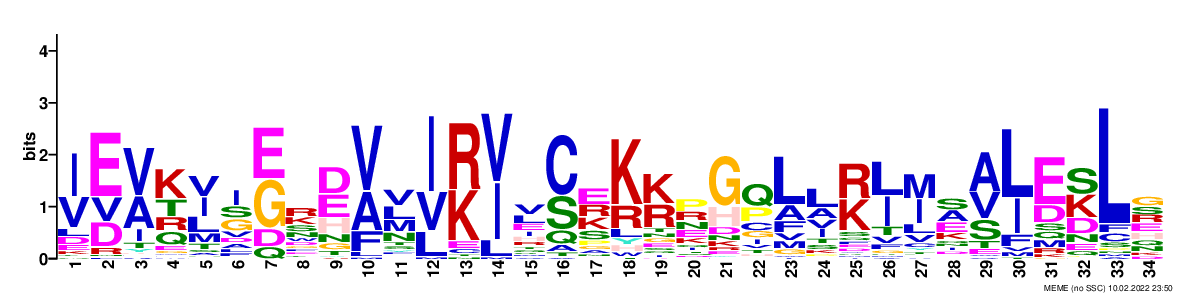


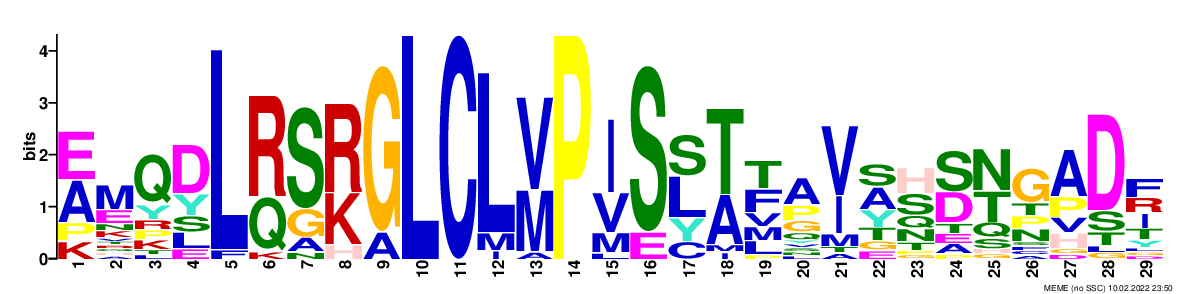


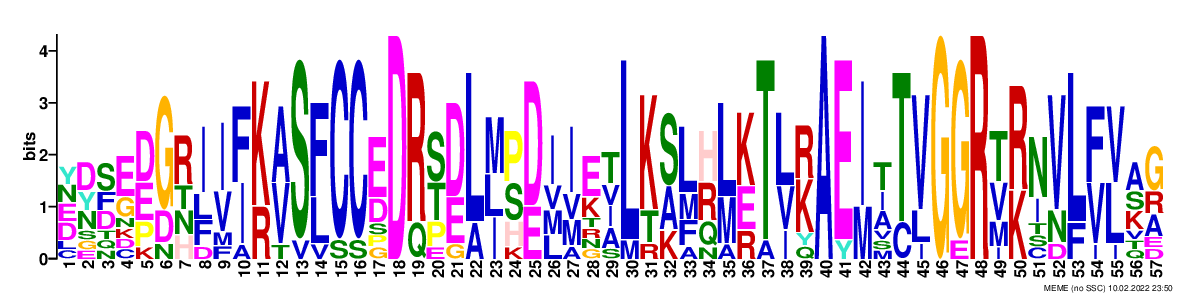


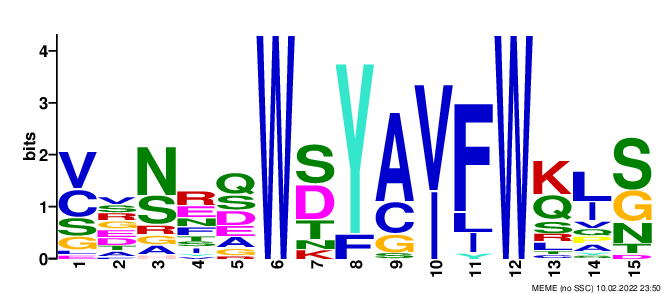


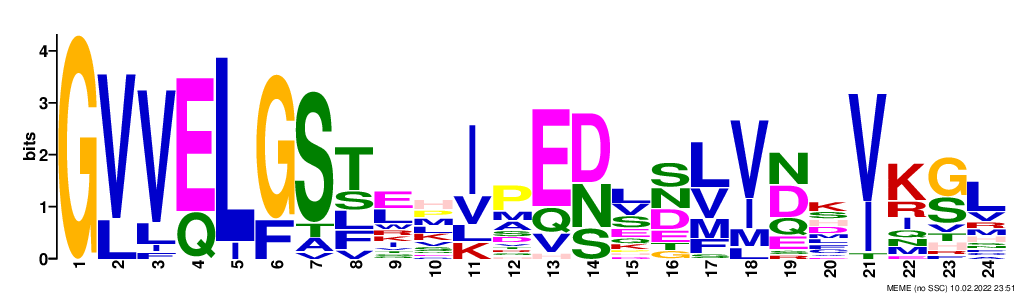


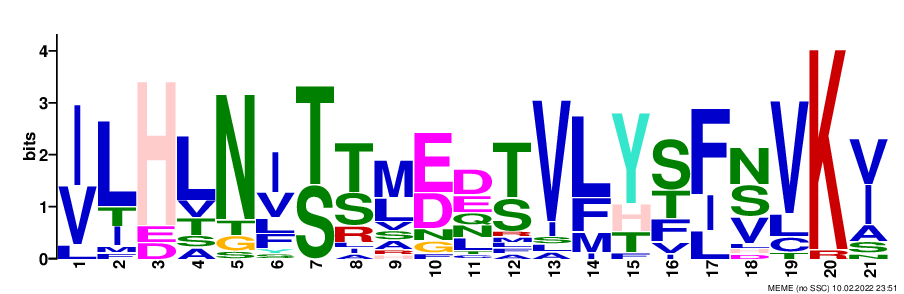


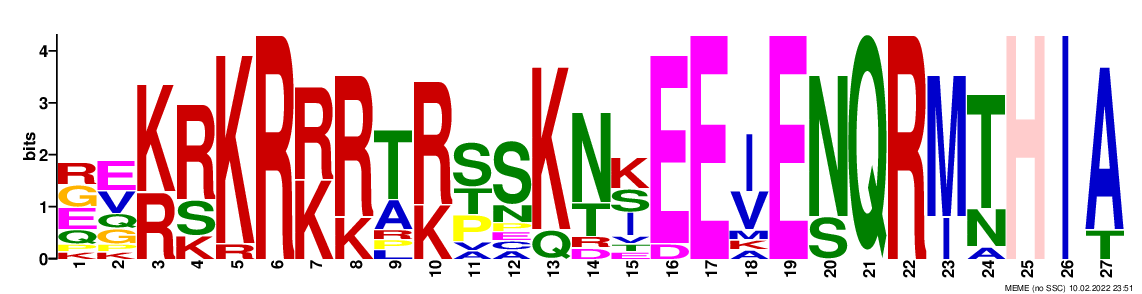


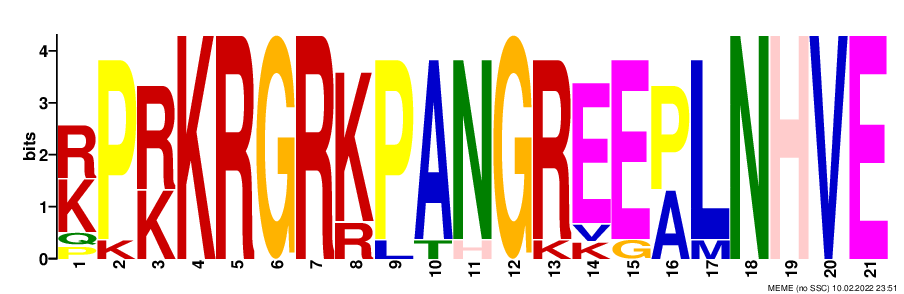


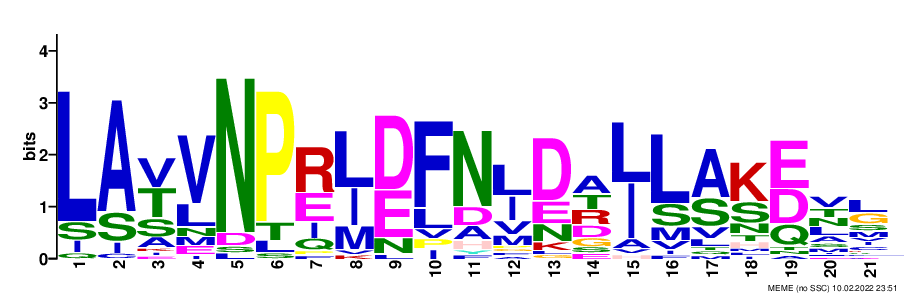


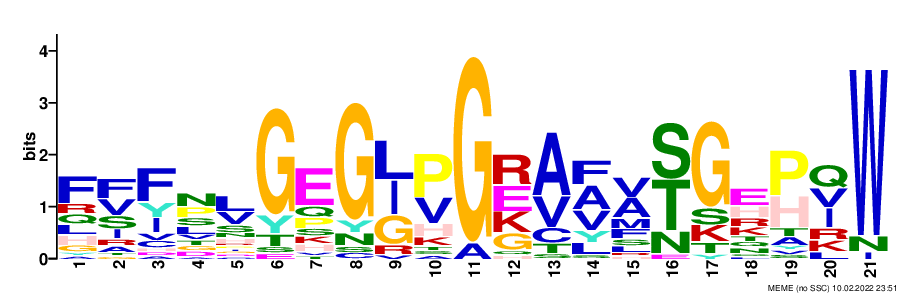


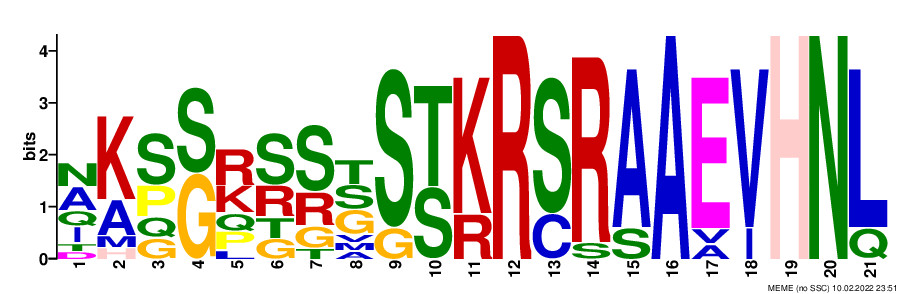


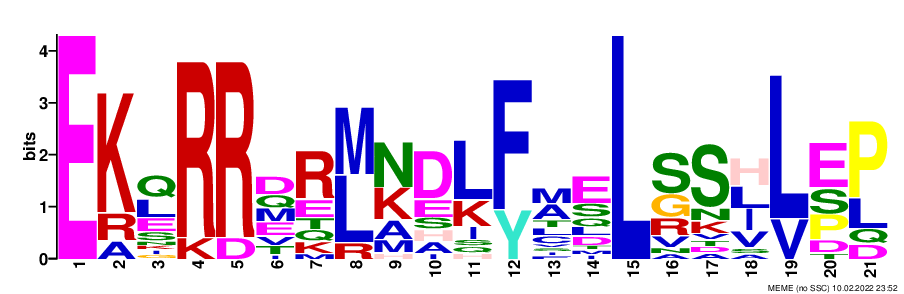


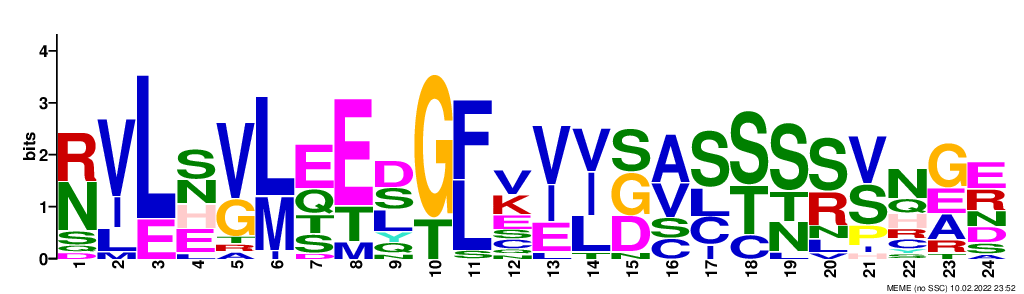


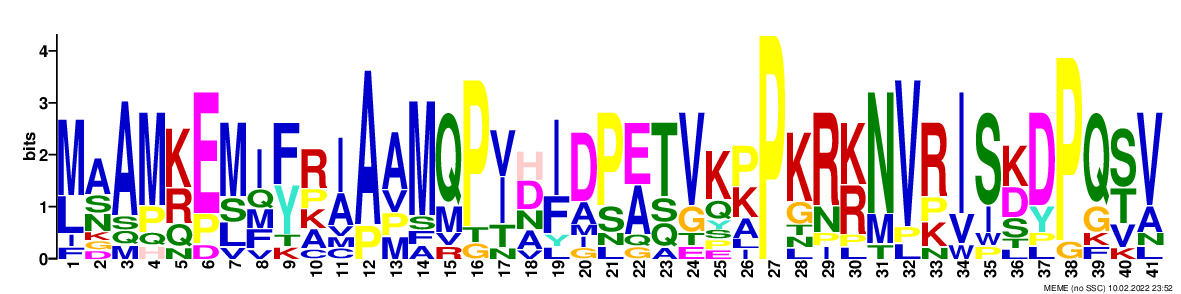


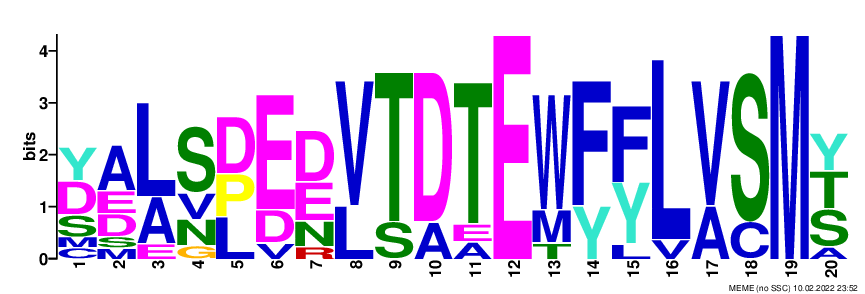

Supplement: Supplementary file 2 — Additional file 2: FigureS1. Sequencelogos of 19 conserved motifs. The height of a letter indicates its relativefrequency at the given position [file 12864_2022_8782_MOESM2_ESM.docx]
