# Supplementary material for: Genome-wide identification and characterization of the bHLH gene family and analysis of their potential relevance to chlorophyll metabolism in Raphanus sativus L
Source: BMC Genomics. 2022 Aug 1;23:548. doi: 10.1186/s12864-022-08782-4 (PMC9344636; doi:10.1186/s12864-022-08782-4)

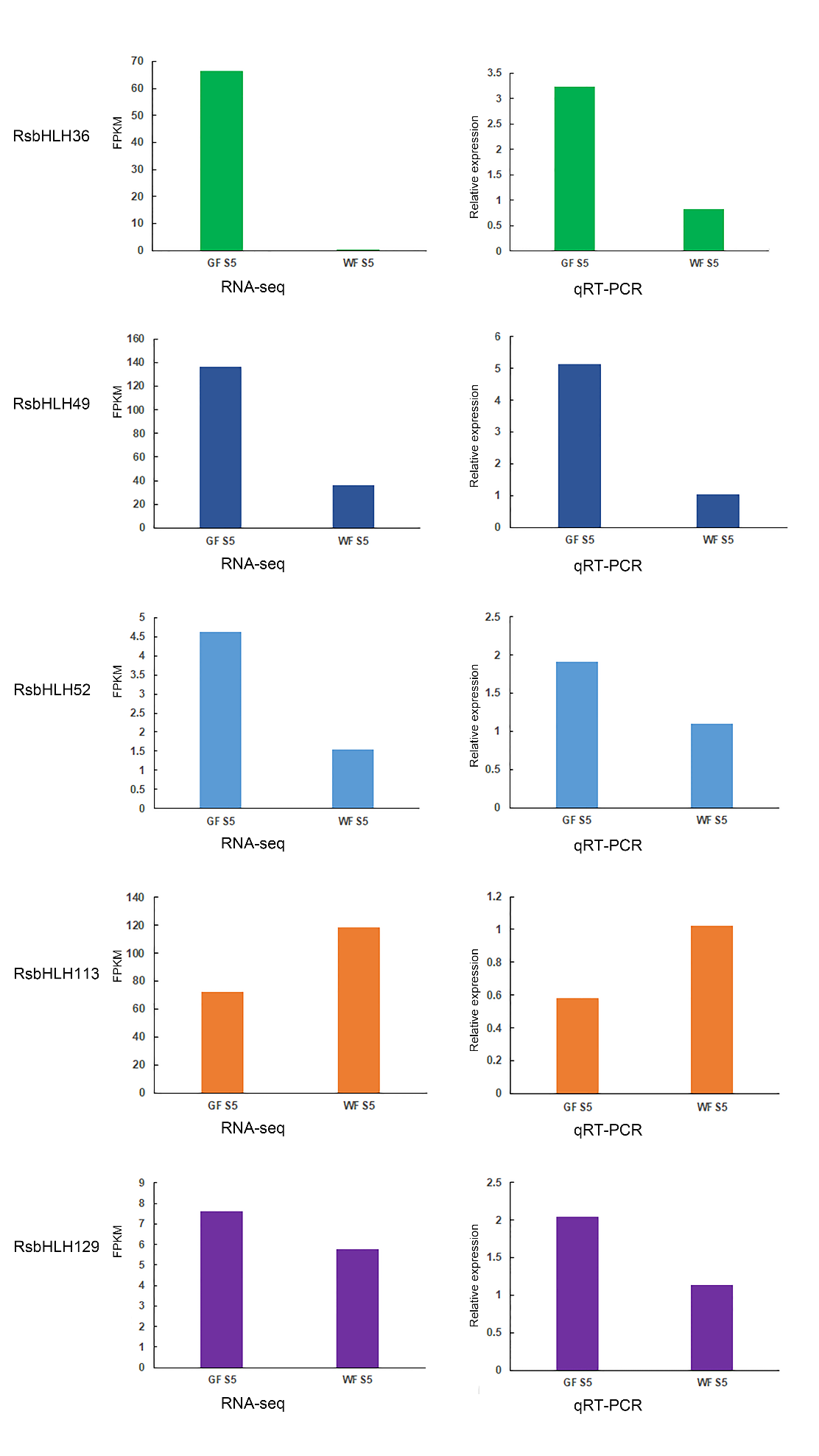
**Figure S2** The expression levels of the fifteen *RsbHLHs* at the fifth stage of GF and WF by qRT-PCR and RNA-seq. qRT-PCR were normalized to the expression of *Actin*.


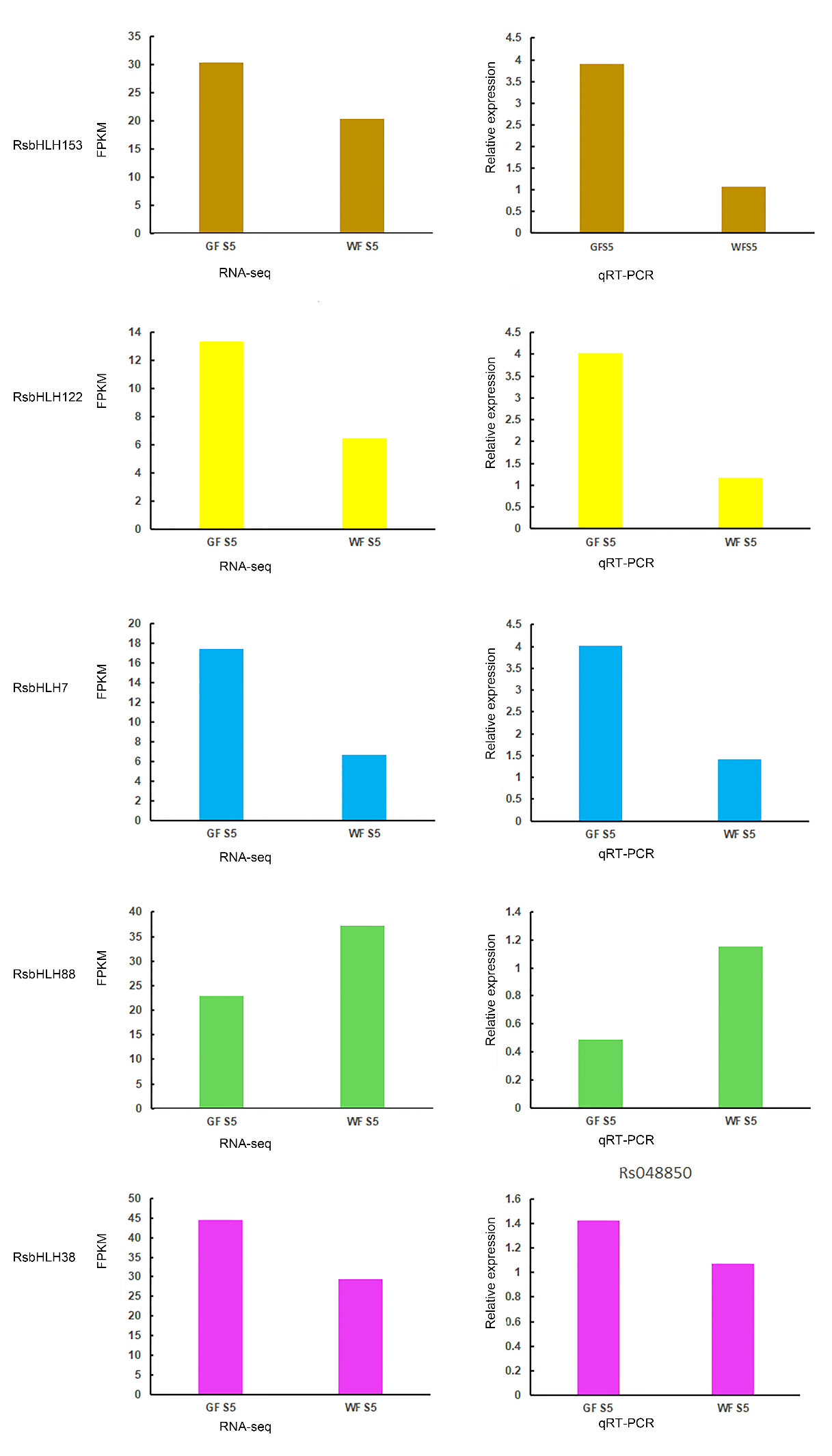


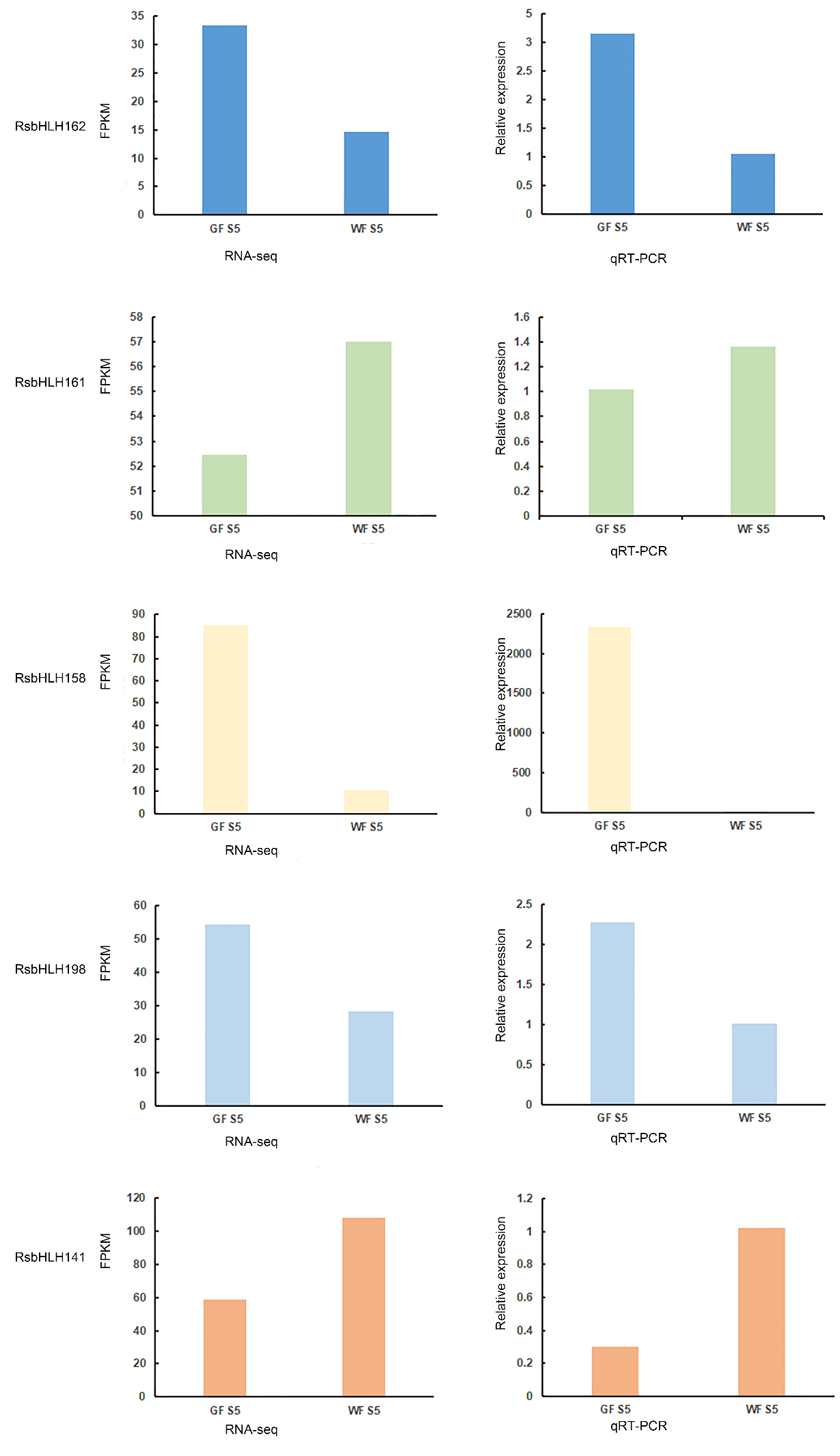

Supplement: Supplementary file 7 — Additional file 7: Figure S2. The expression levels of the fifteen RsbHLHs at the fifth stage of GF and WF by qRT-PCR andRNA-seq. qRT-PCR were normalized to the expression of Actin [file 12864_2022_8782_MOESM7_ESM.docx]
